# Exploring Heart Disease–Related mHealth Apps in India: Systematic Search in App Stores and Metadata Analysis

**DOI:** 10.2196/53823

**Published:** 2025-03-10

**Authors:** Keerthi Dubbala, Roshan Prizak, Ingrid Metzler, Giovanni Rubeis

**Affiliations:** 1 Division of Biomedical and Public Health Ethics Department of General Health Studies Karl Landsteiner University of Health Sciences Krems Austria; 2 Karlsruhe Institute of Technology Karlsruhe Germany

**Keywords:** mobile health apps, mHealth apps, heart disease, data collection methods, natural language processing, metadata analysis, Apple App Store, Google Play Store, mobile phone

## Abstract

**Background:**

Smartphone mobile health (mHealth) apps have the potential to enhance access to health care services and address health care disparities, especially in low-resource settings. However, when developed without attention to equity and inclusivity, mHealth apps can also exacerbate health disparities. Understanding and creating solutions for the disparities caused by mHealth apps is crucial for achieving health equity. There is a noticeable gap in research that comprehensively assesses the entire spectrum of existing health apps and extensively explores apps for specific health priorities from a health care and public health perspective. In this context, with its vast and diverse population, India presents a unique context for studying the landscape of mHealth apps.

**Objective:**

This study aimed to create a comprehensive dataset of mHealth apps available in India with an initial focus on heart disease (HD)–related apps.

**Methods:**

We collected individual app data from apps in the “medical” and “health and fitness” categories from the Google Play Store and the Apple App Store in December 2022 and July 2023, respectively. Using natural language processing techniques, we selected HD apps, performed statistical analysis, and applied latent Dirichlet allocation for clustering and topic modeling to categorize the resulting HD apps.

**Results:**

We collected 118,555 health apps from the Apple App Store and 108,945 health apps from the Google Play Store. Within these datasets, we found that approximately 1.7% (1990/118,555) of apps on the Apple App Store and 0.5% (548/108,945) on the Google Play Store included support for Indian languages. Using monograms and bigrams related to HD, we identified 1681 HD apps from the Apple App Store and 588 HD apps from the Google Play Store. HD apps make up only a small fraction of the total number of health apps available in India. About 90% (1496/1681 on Apple App Store and 548/588 on Google Play Store) of the HD apps were free of cost. However, more than 70% (1329/1681, 79.1% on Apple App Store and 423/588, 71.9% on Google Play Store) of HD apps had no reviews and rating-scores, indicating low overall use.

**Conclusions:**

Our study proposed a robust method for collecting and analyzing metadata from a wide array of mHealth apps available in India through the Apple App Store and Google Play Store. We revealed the limited representation of India’s linguistic diversity within the health and medical app landscape, evident from the negligible presence of Indian-language apps. We observed a scarcity of mHealth apps dedicated to HD, along with a lower level of user engagement, as indicated by reviews and app ratings. While most HD apps are financially accessible, uptake remains a challenge. Further research should focus on app quality assessment and factors influencing user adoption.

## Introduction

### Background

Mobile health (mHealth) apps are prominent among the recommended interventions for addressing health disparities and improving health care accessibility. mHealth, as defined by the World Health Organization, encompasses the use of mobile devices like phones and wireless tools to enhance health care practices, boost health information access, and encourage positive behavioral changes [1,2]. In low-resource settings like India, mHealth offers a cost-effective means to bridge health care gaps [2-8].

India has experienced a substantial surge in mobile phone technology adoption, with more than 70% of urban and 50% of rural households using smartphones [9]. The number of smartphones in India has been estimated to reach 1 billion by the end of 2023 [10]. This widespread use presents a valuable opportunity for leveraging smartphone health apps (mHealth apps) to enhance health care delivery [11-14]. mHealth apps are also being used by community health workers, making them accessible to people without smartphones [15]. During the COVID-19 pandemic, mHealth apps proved particularly valuable by enabling teleconsultations, contact tracing, and web-based prescription services, effectively overcoming barriers to traditional health care access [16-19].

However, mHealth apps can also contribute to increasing systemic inequities. They may risk widening health disparities through a lack of inclusivity and by addressing the health needs of already advantaged groups [20]. Language options in the mHealth apps, or the lack of them, have been highlighted in previous studies [21-25]. In addition to social determinants of health, digital determinants like technology access and community infrastructure (eg, broadband internet) can have a crucial impact on mHealth outcomes and health [25-30]. The COVID-19 pandemic has highlighted how limited access to digital technologies or supporting infrastructures, apps unsuited for target groups, and lack of high-quality data for tailoring interventions to the needs of marginalized groups might undermine the very potential of digital technologies for mitigating health disparities [31]. Therefore, understanding health disparities caused by mHealth apps and creating solutions for working toward health equity is crucial and requires several research efforts.

Although there is limited research on mHealth apps in India, there are many studies on mHealth apps in general. Many are systematic reviews looking at previous literature on individual or small groups of apps [25-30,32]. There are also reviews [4,33] and policy proposals [34] on mHealth apps. Many of these studies focused on the quality, privacy, and security concerns of mHealth apps [35-40]. There are also studies that focused on rating the quality of mHealth apps, such as the one using the MARS (Mobile Application Rating Scale) tool [41,42]. While assessing the quality of apps is essential, this is still manual, time-consuming, and does not give an overall picture of the mHealth apps available. There is a noticeable gap in research that comprehensively assesses the entire spectrum of existing health apps or extensively explores apps for specific health priorities from a health and health care perspective. This gap is even more evident in the context of mHealth apps available in India.

While some studies have investigated the landscape of mHealth apps in app stores, they have used methodologies based primarily on manual searching and screening of apps. Among those, Siddiqui et al [43] focused on orthodontic-related apps, Bassi et al [16] on COVID-19–related apps, and Coulon et al [27] on stress management–related apps. From their search processes, they gathered 952, 4600, and 902 apps, respectively, which were then manually screened and reviewed. However, searching app stores and screening apps manually is a time- and labor-intensive process [27], especially when exploring a large number of apps. Moreover, app store search functions tend to prioritize popular or highly rated apps, which, while useful for analyzing the most widely used apps, limits the ability to gain a comprehensive view of the app landscape. This approach may overlook lesser-used apps that could provide insights into why some apps fail to gain traction.

A significant obstacle in carrying out more extensive studies focusing on a diverse and larger set of mHealth apps is the difficulty of acquiring such a comprehensive dataset. There are some publicly available datasets [35] obtained using large-scale web crawling methods, with information about many apps available on such app stores. However, such datasets do not record all available information for each app and are not usually updated with the latest information. Furthermore, since the data on these datasets are not collected with a specific intention, they may not be entirely suitable for a particular research question. While Danahiswari et al [44] and Fu [45] have used such datasets and additional scraping methods in the context of mHealth apps, their focus was app development and market analysis. Other researchers also used similar scraping methods. In 2015, using web scraping methods, Xu and Liu [46] and Dehling et al [47] made datasets of 24,405 and 60,000 mHealth apps, respectively. More recently, in 2020, Tsinaraki et al [48] analyzed COVID-19–related apps from the 2 app stores using updated scraping methods. However, accessing these app stores, especially Google Play Store, by similar methods has become significantly more challenging due to more restrictive database designs, throttling limits, and IP blocking [48]. Stach et al [35] looked at mHealth apps and proposed a pipeline to search for specific types of apps from the app stores in their crawling techniques, overcoming the restrictions posed by these app stores.

### Objective

Building on the above methods, we aimed to create a comprehensive dataset of mHealth apps using an automated and replicable method for collecting and analyzing metadata from mHealth apps. We present an updated web crawling method for obtaining information about mHealth apps in a particular country. This method can help map the landscape and enable the analysis of mHealth apps. We used these methods to prepare a dataset of mHealth apps available in India from 2022 to 2023 on the Apple App Store and the Google Play Store. As a country with a large and diverse population, India faces unique health care challenges that can be effectively addressed through innovative technological solutions [4,49]. By exploring the landscape of mHealth apps, we can gain insights into their effectiveness and relevance and identify gaps and potential for improvement.

As a crucial first step in mapping these apps, we concentrated on apps relevant to heart disease (HD) using natural language processing (NLP) and clustering techniques. HD cause the most significant number of deaths in India and, therefore, are a crucial health priority to focus on [49]. This evidence can serve as a foundation for shaping health care policies, improving access, and promoting positive health outcomes.

## Methods

### Overview

The methodology in this study was developed to address the need for a comprehensive and updated dataset of apps available in India, with a focus on HD apps. Our approach builds on previous studies that have used automated methods for collection of metadata from mHealth apps [27,42-46] and aimed to overcome the challenges posed by restrictive database designs, throttling limits, and IP blocking by using a replicable web crawling method. We used analysis techniques, such as NLP and clustering, which have been used in health care research for various applications [50-54]. These techniques enabled us to identify apps related to HD and group these apps further on the basis of their focus and utility. Our methodology facilitates the effective gathering of a comprehensive dataset and allows for the process to be scaled and adapted for future research.

**Figure 1:**
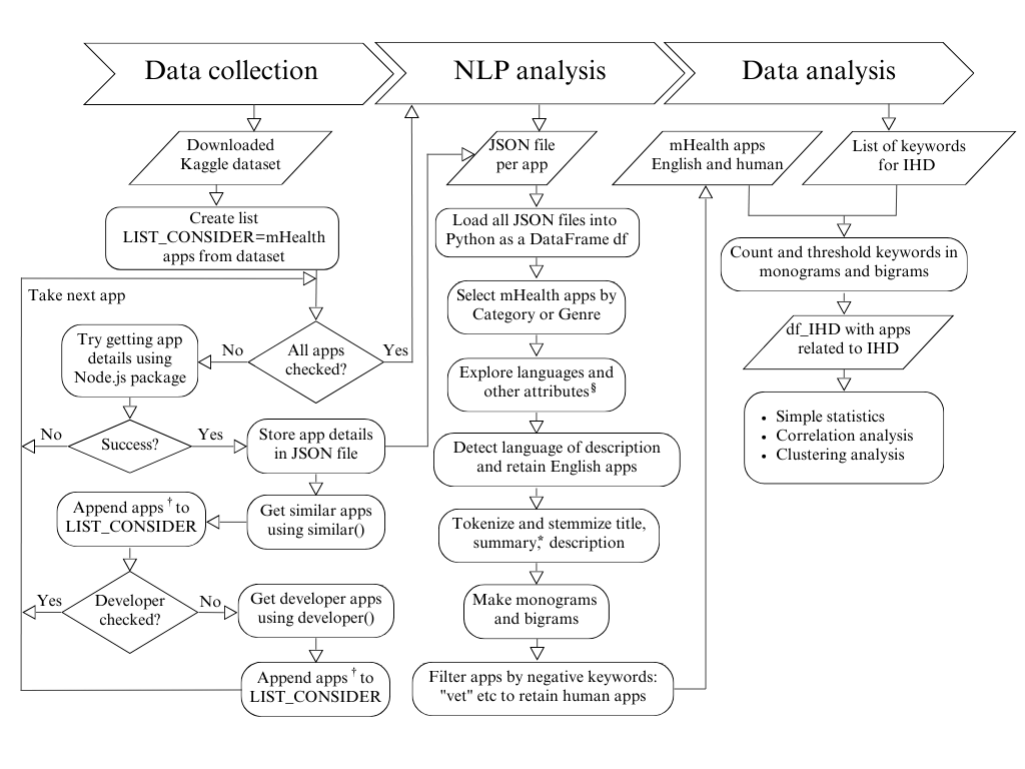
Methodology for extracting mobile health (mHealth) apps and selecting and analyzing apps for heart diseases. †While for Apple App Store, at this stage we already retain only mHealth apps; §Other attributes include installs, app sizes, ratings, reviews, rating scores; *Apple App Store apps do not have summary included. IHD: ischemic heart disease; NLP: natural language processing.

In this section, we will first discuss our data collection methodology. Then, we will discuss the NLP methods we used for identifying HD apps, followed by statistical analysis and clustering of selected apps. Figure 1 illustrates all the steps included in this methodology.

### Data Collection

The 2 final datasets used for analysis contained app metadata from the “medical” and “health and fitness” categories on the Google Play Store in December 2022 and on the Apple App Store in July 2023, providing a snapshot of the app stores in these 2 months. This metadata includes app attributes, such as a unique app ID, the app’s title, the provided description, the number of installs, and the price of the app, among other such details. A complete list of attributes is included in Table 1.

**Table 1:** Attributes of the apps in the datasets.

Attribute description	Attribute of the apps from the Apple App Store	Attribute of the apps from the Google Play Store	Attribute used for
Unique identification	ID	ID	Identifying individual apps and preventing duplicates during data collection.
App name	Title	Title	Tokens, monograms, and bigrams
App summary	N/A^a^	Summary	Tokens, monograms, and bigrams
App description	Description	Description	Tokens, monograms, bigrams, and number of characters (description length)
Release date	Released	Released	Tracking number of apps released over the years
App size	Size	Size	Average size of apps on each platform
Required operating system	Required_os	Android_2022	Most used versions of operating systems
Price of the app	Price	Price	Price of apps in INR^b^
Rating-score (ie, star-rating)	Score	Score	Average rating-scores
Number of reviews	Reviews	Reviews	Average number of reviews
App’s category	Category	Category	To select health and medical apps
Presence of in-app purchases	N/A	IAP^c^	Number of apps with in-app purchases
Number of ratings	N/A	Ratings	Average number of ratings
Number of installs (ie, downloads)	N/A	installs_2022	Average number of installs
All the languages the app is available in	Languages^d^	—^e^	Distribution of languages in apps
Updated date	Updated	✓^f^	Tracking the number of apps updated over the years

^a^N/A: not available.

^b^INR: Indian national rupee.

^c^IAP: in-app purchases.

^d^Apple App Store lists many languages (ie, those in which the app can be used) in the languages attribute of a given app.

^e^Google Play Store does not have an attribute called languages. In both cases, for filtering apps based on language, we detected the language of the app description. This is illustrated in detail in Multimedia Appendix 1.

^f^Google Play Store does not have the attribute for updated year at the time of data collection.

We obtained the initial datasets from (1) a Kaggle dataset [55] containing >2.3 million apps scraped from the Google Play Store in June 2021 and (2) a Kaggle dataset [56] containing >1.2 million apps scraped from the Apple App Store in October 2021.

As Google Play Store has changed its internal data structure, it has become extremely challenging to use crawling tools to scrape its whole database. In such a context, the publicly available Kaggle datasets from recent years offer a great starting point to expand and compile the latest dataset. As these datasets are publicly available, researchers can obtain them as starting points for future efforts to expand such datasets or already perform analysis on them. An important note is that the Kaggle datasets do not have all the necessary information like app descriptions, which we therefore obtained through our scraping methods as follows.

For each of the app stores, we extracted app-specific unique identifiers (ie, *app_id*s) to create 2 separate lists called LIST_CONSIDER, comprising apps from the “medical” and “health and fitness” categories.

We then used the *Node.js* modules *google-play-scraper* [57] and *app-store-scraper* [58] to access updated app details from the Google Play Store and the Apple App Store in December 2022 and July 2023, respectively, and to expand our dataset of “medical” and “health and fitness” apps. For each *app_id* in LIST_CONSIDER, we performed the following three steps:

(1) We queried the app’s metadata and stored the returned metadata in a JSON file named after its *app_id*. If the app was not available on the queried date, we skipped the next 2 steps and considered the next app in LIST_CONSIDER.

(2) We obtained a list of other apps deemed as “similar” to the app using the “similar()” function. This function uses an internal algorithm to suggest similar apps given an input app. From this list of apps, we filtered to retain only “medical” and “health and fitness” apps and added their corresponding *app_id*s to the end of LIST_CONSIDER.

(3) We obtained a list of other apps developed by the same developer of the app using the “developer()” function. We again filtered this list of apps to retain only “medical” and “health and fitness” apps and added their corresponding *app_id*s to the end of LIST_CONSIDER.

As we kept adding more apps to LIST_CONSIDER, as described above, our dataset grew. We repeated this process until LIST_CONSIDER was exhausted, and no new *app_id*s could be added. In the end, we had 2 sets of JSON files—one for the Google Play Store and the other for the Apple App Store.

### Filtering Medical and Health and Fitness Apps

We analyzed these 2 sets of JSON files as separate datasets (ie, Google dataset and Apple dataset). The 2 datasets were never added together into a single analysis but only compared against each other. To start with, we used 2 steps of filtering as follows: (1) the main language used in the description should be English, and (2) the app should be concerned with human health and not with animal health. To achieve the first filtering step, we detected the main language of the description using the *langdetect* package in Python and selected only those with descriptions in English language. This step was important to use NLP techniques later. Then, from the resulting English language apps, we removed veterinary and animal health apps using NLP techniques. To identify apps related to animal health topics, we looked for the presence of a preselected set of veterinary and animal health keywords in the app title, summary (ie, present only in the Google dataset), or description. We discuss these techniques in detail in the section for selecting apps for HD.

### Selecting Apps Related to HD

NLP applies computational techniques to analyze natural language and speech [59]. NLP treats textual content as data and offers a variety of methods for efficiently processing substantial amounts of text [60]. In this study, we used the app titles, app summaries (ie, present only in the Google dataset), and app descriptions as our text data. We used tokenization, lemmatization, stop-word removal, and n-grams as part of our NLP methodology [60]. Tokenization identifies individual words or tokens within a given text. Lemmatization transforms each word into its root form by removing suffixes. For instance, lemmatization consolidates word forms like “diagnose,” “diagnosis,” “diagnosed,” and “diagnosing” into the common root word “diagnose.” By treating different word forms as a single entity, lemmatization enables accurate analysis in further steps. After eliminating stop-words like “and,” “or,” “also,” and “then” as well as special characters, we made monograms and bigrams from the text. Monograms are single words, while bigrams consist of pairs of consecutive words. These allow for the analysis of not only individual words by themselves but also in the context of their use. For example, the words “heart” and “disease” each have some meaning in the health context, whereas “heart disease” as a pair conveys a specific meaning. Using monograms and bigrams allows us to capture and use both contexts in our analysis.

We made a “dictionary” of terms relevant to HD using Medical Subject Headings terms from PubMed database [61] and added more terms from Google searches. This dictionary is listed in Table 2.

**Table 2:** Heart diseases—keyword dictionary.

Categories	Keywords
Diseases	heart disease, cardiovascular disease, coronary artery disease, myocardial infarction, ischemic heart disease, angina, atherosclerosis, hypertensive heart disease, valvular heart disease, congenital heart disease, rheumatic heart disease, heart failure, cardiomyopathy, arrhythmia, atrial fibrillation, ventricular fibrillation, supraventricular tachycardia, bradycardia, heart murmur, pericarditis, endocarditis, myocarditis, Kawasaki disease, systemic lupus erythematosus, rheumatoid arthritis, giant cell myocarditis, viral myocarditis, dilated cardiomyopathy, hypertrophic cardiomyopathy, restrictive cardiomyopathy, blocked arteries, clogged arteries, heart blockage, heart attack, and cardiac arrest
Symptoms	chest pain, shortness of breath, fatigue, edema, high blood pressure, hypertension, palpitations, chest discomfort, chest tightness, heartache, breathlessness, labored breathing, heavy breathing, racing heart, rapid heartbeat, irregular heartbeat, skipped beats, swelling, and fluid retention
Treatments or procedures	coronary artery bypass graft, percutaneous coronary intervention, stent, echocardiogram, electrocardiogram, cardiac catheterization, cardiac magnetic resonance imaging, cardiac rehabilitation, bypass surgery, heart surgery, open-heart surgery, coronary bypass, bypass surgery, stent surgery, stent placement, balloon procedure, valve surgery, heart valve repair, valve replacement, cardiac rehabilitation, heart transplant, new heart, and artificial heart
Medications	beta-blockers, calcium channel blockers, angiotensin-converting enzyme inhibitors, diuretics, statins, antiplatelet medications, anticoagulants, angiotensin receptor blockers, blood thinners, clot-busting drugs, clot-preventing medication, blood pressure medicines, and cholesterol-lowering drugs
Medical devices	pacemaker, defibrillator, shock device, and electrical device
Medical specialties	cardiology, cardiologist, and cardiothoracic

We made monograms and bigrams from this HD keyword dictionary (ie, HD monograms and bigrams). We also made monograms and bigrams for each app’s title, summary, and description (ie, app monograms and bigrams). Then, for each app, we counted the frequency of HD monograms and bigrams in the app monograms and bigrams, which we used to select apps relevant to HD. We tried different iterations and found that selecting apps with (1) at least 1 *relevant* HD monogram or (2) at least 1 HD bigram is an optimal method. We eliminated apps with only one monogram if the monogram was not exclusive to HD—for example, “pain.” However, apps with single and HD-relevant monograms, such as “ECG” (electrocardiogram), were selected. Apps without any HD monograms or bigrams in their app monograms and bigrams were not selected.

### Simple Statistics and Correlation Analysis of HD Apps

We explored the HD apps using various app attributes like app sizes, app prices, number of ratings, rating-scores, number of reviews, presence of in-app purchases, the release year of each app, and the length of description. We used descriptive statistics like arithmetic mean to understand the distribution of the apps along the mentioned attributes. We also performed correlation analysis to understand the relationships among these attributes.

### Clustering and Topic Modeling of HD Apps

To understand the different types of apps present in the selected HD apps, we performed cluster analyses. We used the latent Dirichlet allocation (LDA) algorithm for clustering and topic modeling using app descriptions as input. This technique allows us to uncover underlying topics within the dataset. We used the *CountVectorizer* function from the *scikit-learn* package to convert the description text into a matrix of token counts. The matrix consists of the counts of the frequency of individual words (ie, tokens) across all the app descriptions. This matrix was then subjected to LDA using the *LatentDirichletAllocation* function from *scikit-learn*. After several iterations using different parameters, we configured the LDA model to identify 3 topics (ie, clusters) using the following parameters: *n_components*=3, max_iter=5, *learning_method*=*'online'*, and learning_offset=50. Each app’s cluster assignment was determined based on the topic with the highest probability in its distribution.

We explored the resulting clusters, but we did not find any further meaningful subclusters. To address specific research inquiries, alternative supervised topic modeling methods could be explored to extract more nuanced subclusters. We also experimented with k-means clustering [44], but our results indicated that LDA clustering yielded superior outcomes with this dataset. Clouds from k-means clustering are attached in Multimedia Appendix 2.

### Tools Used

In general, we used *Python* as the main scripting language, the *pandas* [62] package for data analysis, and *matplotlib* [63] for data visualization. To expand the list of apps by crawling and scraping the app stores, we used *Node.js*—modules “*google-play-scraper*” [57] and “*app-store-scraper*” [58] for the Google Play Store and the Apple App Store, respectively. We wrapped the *Node.js* scripts inside a *Python* script that iteratively looped over the Kaggle dataset and expanded the list of apps with their details. Next, for NLP tasks like tokenization and stemmization, we used the *nltk* [64] package. For language detection, we used the *langdetect* [65] package and for word cloud visualizations, we used the *wordcloud* [66] package. For further NLP analysis, we used vectorizers and methods from the *scikit-learn* package, and for LDA visualization, we used the *pyLDAvis* [67] package.

The code written and used for data collection and analysis in this study is available in our GitHub repository [68].

### Ethical Considerations

No ethics board review was needed as our study does not use patient data or involve human participants.

## Results

### Overview

**Figure 2:**
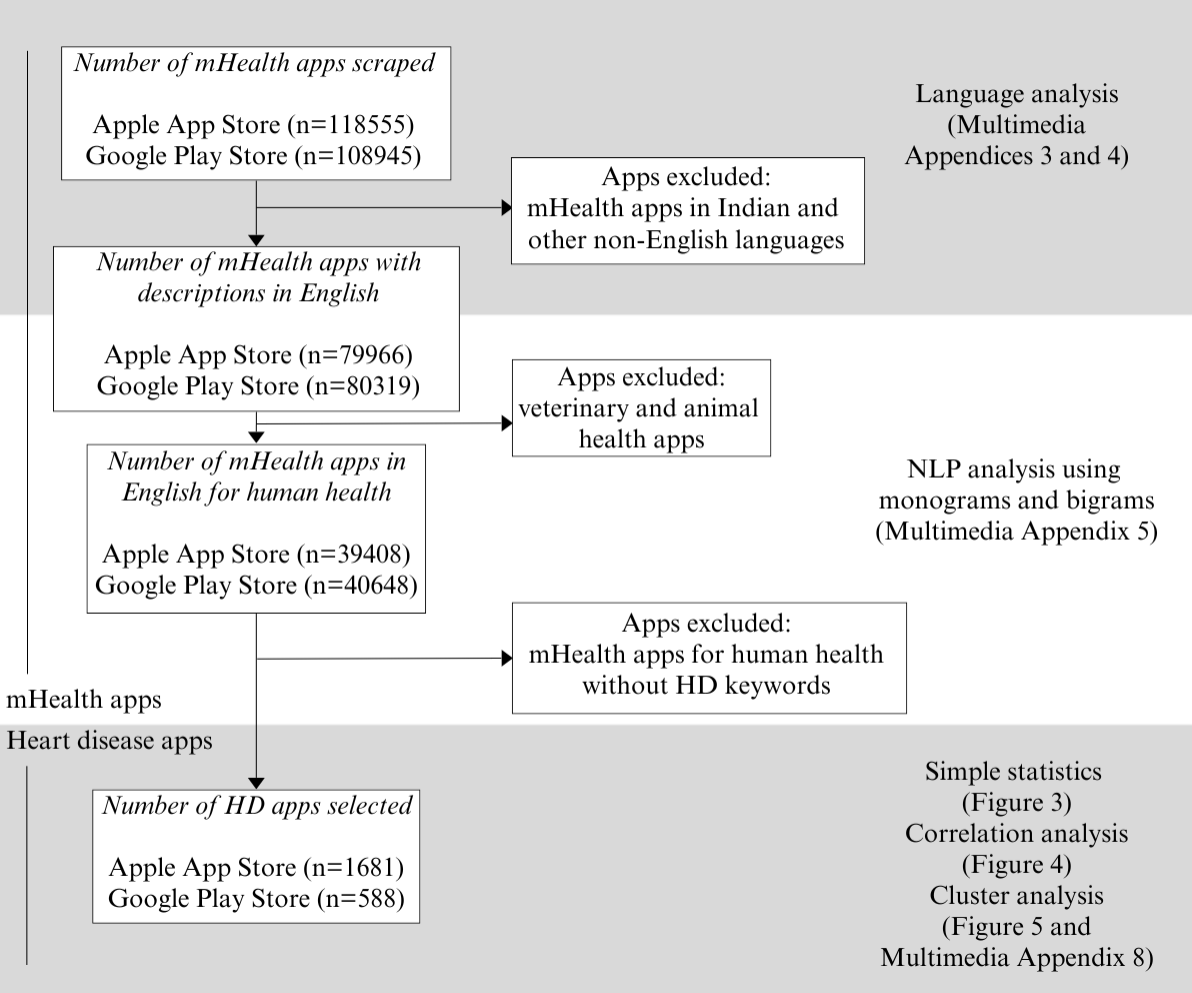
Summary of results. HD: heart disease; NLP: natural language processing.

Using the data collection methods described in the previous section, we scraped 118,555 apps from the Apple App Store and 108,945 apps from the Google Play Store categorized as “health and fitness” or “medical” (ie, health and medical apps). Figure 2 illustrates a summary of the results discussed below.

### App Language

An important consideration here is the presence of the app attribute *language* in the Apple App Store dataset, and its absence in the Google Play Store dataset. Apple App Store’s *language* attribute lists all the languages an app supports and is exactly the information needed to evaluate the language support of an app. However, the Google Play Store dataset lacks this attribute, so it is not possible to know which languages it supports. Hence, as an indirect indication, we used the main language of the app description detected by the *langdetect* package as Google Play Store’s stand-in attribute for languages.

In the resulting health and medical apps, Apple App Store has more than 90% (109,425/118,555, 92.3%) apps that support English whereas Google Play Store has 73.72% (80,319/108,945) apps in English (ie, descriptions in English). We found that approximately 1.7% (1990/118,555) of apps on the Apple App Store included support for Indian languages and 0.5% (548/108,945) of apps (ie, descriptions) on the Google Play Store were in Indian languages. Among Indian languages, apps supporting Hindi makes up the majority on Apple App Store whereas apps in Bengali are the majority on Google Play Store followed by Hindi. Although there were only a few health and medical apps on Google Play Store in Indian languages, these apps had higher average rating-scores, installs, and higher average number of reviews compared to others. The distribution of apps in different languages and the average rating scores and number of installs and reviews of apps in Indian languages are presented in Multimedia Appendices 3 and 4, respectively.

As most of the apps were in English language, we continued with these apps for further analysis, excluding apps in Indian and other non-English languages. After screening for apps with descriptions in the English language, and removing veterinary and animal health apps, we had 39,408 apps from the Apple App Store and 40,648 apps from the Google Play Store.

### HD Apps

Using HD monograms and bigrams, we selected 1681 HD apps from the Apple App Store and 588 HD apps from the Google Play Store. These HD apps had elevated frequencies of various HD monograms and bigrams as expected. A list of frequently occurring keyword monograms and bigrams in selected HD apps are shown in Table 3 and the corresponding word clouds are presented in Multimedia Appendix 5.

**Table 3:** Keyword monograms and bigrams found in (selected) heart disease apps.

Store		Keywords
**Apple App Store**		
	Monograms	drug, beat, race, heart, cardiovascular, block, rapid, cardiac
	Bigrams	chest pain, cardiac arrest, heart attack, short breath, supraventricular tachycardia, valvular heart, skip beat, irregular heartbeat
**Google Play Store**		
	Monograms	heart, blood, pain, cardiovascular attack, chest, short, cardiac
	Bigrams	heart attack, chest pain, cardiac arrest, irregular heartbeat, chest discomfort, valvular heart, supraventricular tachycardia, rapid heartbeat

### Exploring HD Apps

**Figure 3:**
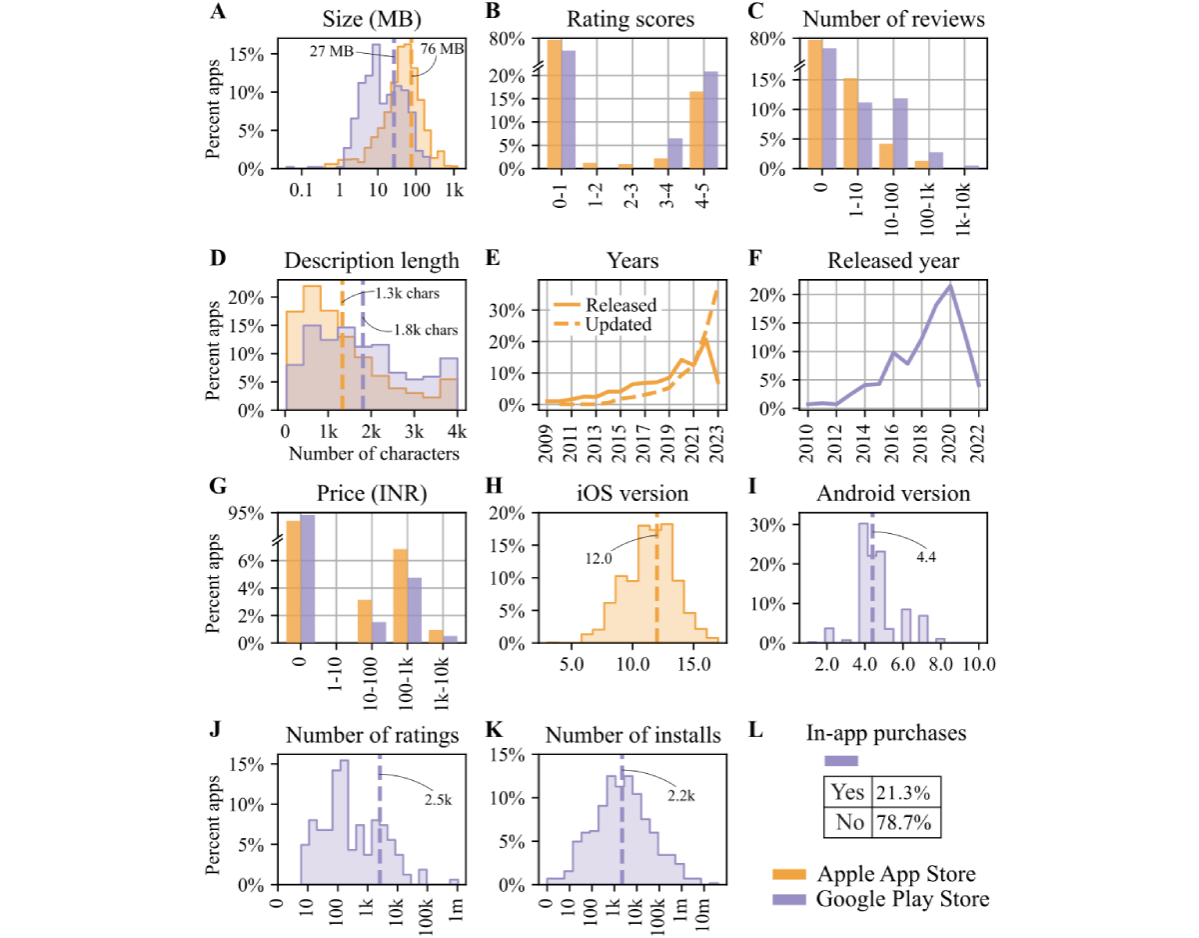
Analysis of attributes for heart disease apps on Apple App Store (N=1681) and Google Play Store (N=588).

The distributions of various app attributes for HD apps from the Apple App Store and the Google Play Store are shown in Figure 3. There had been an increase in the number of HD apps released over the years on both the Apple App Store and the Google Play Store. Many of the Apple App Store HD apps had been updated in the last year (Figures 3E and 3F). The average size of HD apps was 76 MB on the Apple App Store and 27 MB on the Google Play Store (Figure 3A). Very few apps had rating-scores, and when they did, they mostly had scores>3 (Figure 3B). Similar to rating-scores, very few apps had reviews (Figure 3C). Among those apps with reviews, there were rarely >1000 reviews. Data for the number of ratings and installs were available only for Google Play Store apps. Many apps did not have any ratings or installs. Among the apps with number of ratings and installs, the average was around 2000 (Figures 3J and 3K).

About 90% (1496/1681 on Apple App Store and 548/588 on Google Play Store) of the HD apps were free on both platforms. Among those apps that were not free, prices were mostly between 100 and 1000 INR (Indian national rupee; US $1.2 and US $12; Figure 3G). About 20% (125/588, 21.3%) of the HD apps on the Google Play Store had in-app purchases (Figure 3L). In-app purchase data was not available for Apple App Store HD apps.

Google Play Store HD apps had an average of 1800 characters and Apple App Store HD apps had 1300 characters in their descriptions (Figure 3D). Many HD apps did not need the latest operating systems (OS) and were functional with earlier OS versions (Figures 3H and 3I).

### Correlation Analysis

**Figure 4:**
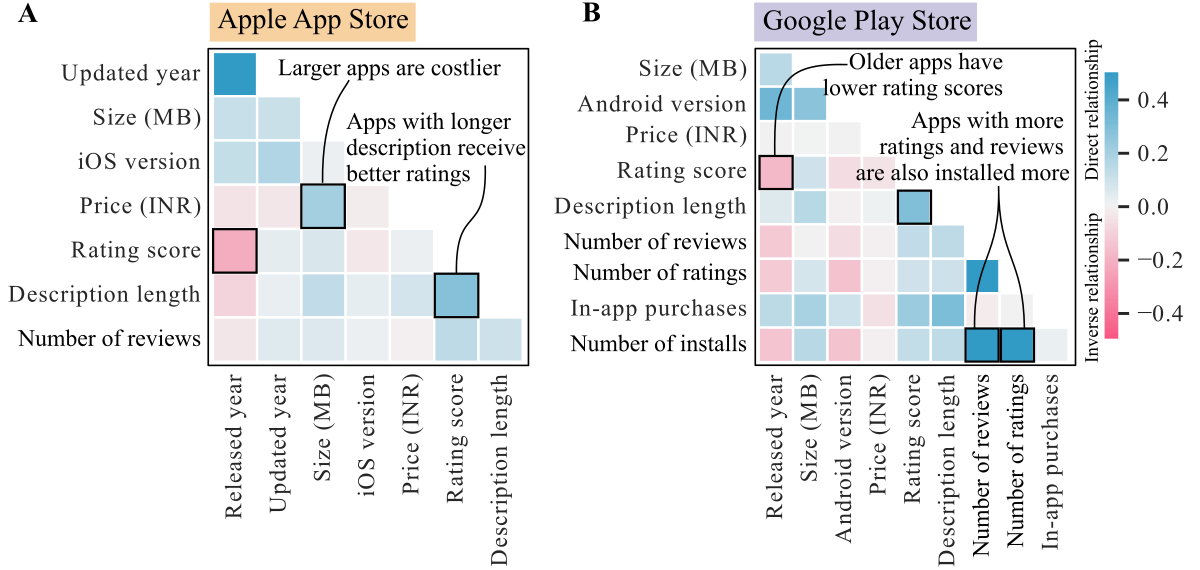
Explorative correlation analysis between the pairs of app attributes.

Figure 4 shows the correlations between pairs of attributes for Apple App Store and Google Play Store HD apps as heatmaps. Correlation matrices are included in Multimedia Appendices 6 and 7. The number of installs for HD apps on Google Play Store was positively correlated with the number of ratings and the number of reviews. On both Apple App Store and Google Play Store, we found a positive correlation between the length of description and rating-scores, and a negative correlation between app release year and rating-score. For HD apps on the Apple App Store, there was a positive correlation between the size and price of the apps.

### Cluster Analysis of HD Apps

**Figure 5:**
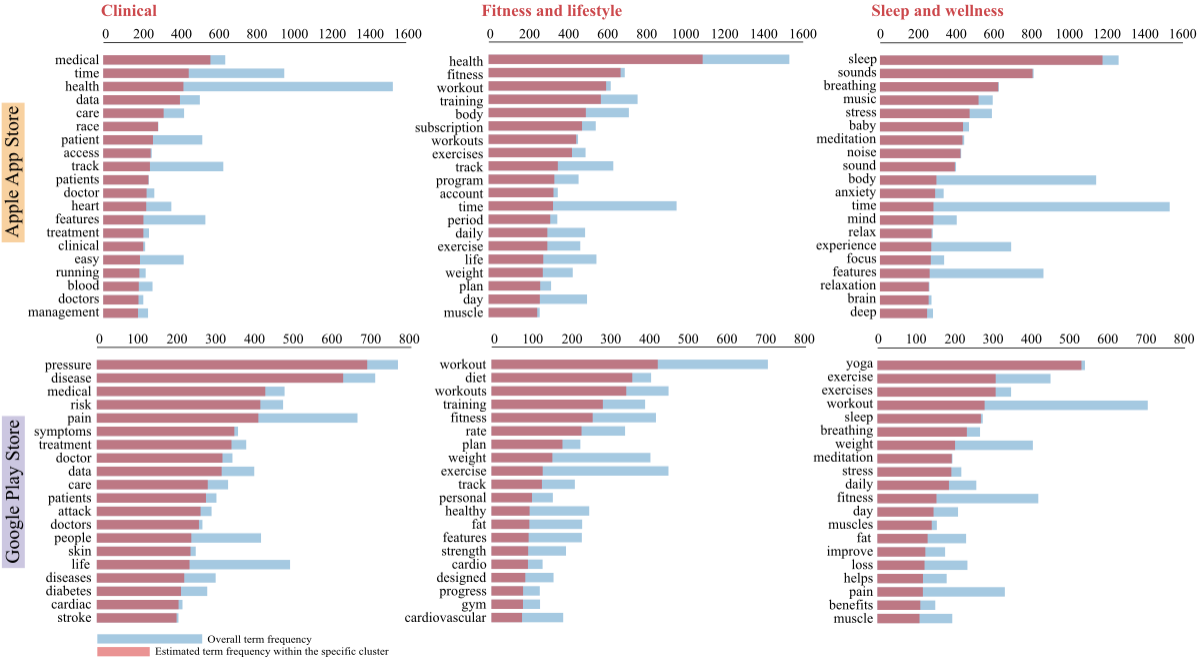
Top 20 most relevant terms by clusters from latent Dirichlet allocation.

Using the LDA topic modeling methods, we identified 3 distinct clusters in both Apple App Store and Google Play Store HD apps. After investigating the topics they dealt with, we assigned them the following names: clinical cluster, fitness and lifestyle cluster, and sleep and wellness cluster. Table 4 shows the number of apps in each cluster and its most relevant terms in app descriptions. An expanded set of top 20 relevant terms in each cluster, their frequency within clusters and their overall frequency in all apps are presented in Figure 5. LDA modeling results using the *pyLDAvis* package and the resulting word clouds for each cluster are included in Multimedia Appendix 8.

Because these clusters were made from apps in 2 categories (ie, “medical” and “health and fitness”), we also checked the distribution of the categories within the clusters (Multimedia Appendix 9). We found that the clinical cluster had many apps from “medical” category. However, it was not exclusive to “medical” category apps. Likewise, apps categorized as “medical” were also found in the other 2 clusters, albeit significantly less in number.

**Figure 6:**
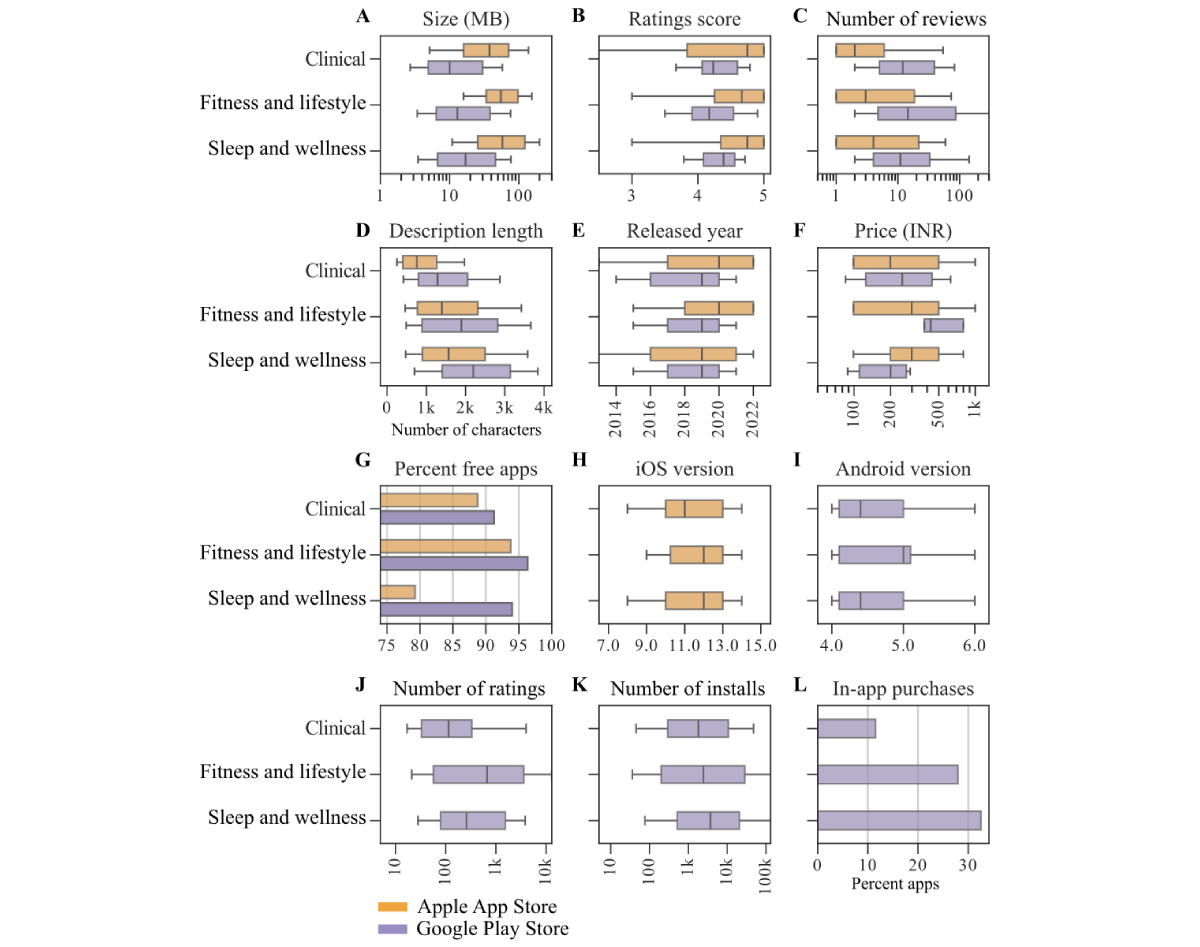
Distributions of various attributes (ie, metadata) of heart disease apps grouped by clusters.

We explored the distribution of app attributes for each of these clusters separately (Figure 6), similar to the analysis above on all HD apps. We found that clinical apps were comparatively smaller in size and had shorter descriptions. Price was higher for apps in the fitness and lifestyle cluster.

**Table 4:** Number of apps in the various clusters from latent Dirichlet allocation cluster analysis.

Cluster name	Number of Apple App Store HD^a^ apps (N=1681), n (%)	Number of Google Play Store HD apps (N=588), n (%)	Most relevant terms for each topic
Clinical	817 (48.6)	286 (48.63)	Disease, medical, cardiac, risk, pain, treatment, and doctor
Fitness and lifestyle	594 (35.33)	136 (23.12)	Diet, workout, training, and fitness
Sleep and wellness	270 (16.06)	166 (28.23)	Sleep, yoga, meditation, and stress

^a^HD: heart disease.

## Discussion

### Languages in Health and Medical Apps

The language diversity within health and medical apps is a notable concern based on our findings. In India, the availability of health and medical apps in Indian languages remains limited, representing only a small fraction of the total. Around 64% of internet users from rural India and 25% of urban India access the internet in their regional language [21]. This shows that most of the mHealth apps that are available in India are not accessible to those who do not understand English, or prefer a regional language.

Previous research has identified the lack of available health apps in native languages in India as a major access barrier. In a study conducted to understand the acceptability of app-based interventions in India, Sinha et al [21] found that language was a significant barrier to adopting health apps, as most of them are available in English. The study by Singh et al [22] focused on privacy policies and directly assessed app descriptions of mental health apps in app stores. Their findings suggest that the lack of native language translation of app description negatively affects the ability of users to understand and evaluate privacy policies.

Outside of India, Huang et al [23] performed a screening of diabetes self-management apps on the Google and Apple app store platforms. They found that most diabetes self-management apps only had English-language descriptions, thereby making them inaccessible to non–English speakers. Haggag et al [24] looked at the mHealth app user reviews on Apple and Google app stores in Australia, the United States, and the United Kingdom. They found that the users faced considerable challenges when they were not able to use the apps in their native language. In a systematic review conducted to study the health equity considerations in mHealth interventions for caregivers of older adults, Garnett et al [25] found that only 11% of app providers considered the provision of alternate language options. These numbers are much worse in Indian mHealth apps. However, despite being fewer in number, apps in Indian languages seem to have better user engagement, as indicated [47] by their higher installs, average ratings, and number of reviews compared to others. This highlights a strong case for developing more health apps in Indian languages.

### Health Priorities: Number of HD Apps

A crucial requirement for mHealth apps is to address existing health issues [69]. Our findings indicate that this is only partly the case in India. Compared to the thousands of apps available in India in the “medical” and “health and fitness” categories, the number of relevant apps for HD, which is number one in the top 10 causes of death in India [49], is exceedingly low. In a scoping study on mHealth initiatives in India in 2016, Bassi [70] searched the Google Play Store and the Apple App Store and found 81 and 107 apps, respectively for HD. Although the numbers have multiplied over the years, the fraction of apps for HD is still small.

While the selection method could be improved to find more apps, if the apps do not have some of the basic keywords, it would be just as difficult for a typical smartphone user to find them as it was for us. Moreover, if the app is not easy to find, it can add to the accessibility barrier from a technological perspective.

We found that the Apple App Store had more HD health and medical apps compared to the Google Play Store, in line with previous research. Dehling et al [47] found that the ratio of Apple mHealth apps to Google mHealth apps was about 9 to 1. However, the numbers for overall mHealth apps in our study (ie, after screening for apps in English language and eliminating animal health-related apps) were comparable on both platforms.

### App Size

We found that the average size of HD apps on the Apple App Store and on the Google Play Store in this study was higher compared to previous studies on mHealth apps. Coulon et al [27] reported that the average size of apps for stress management on the Apple App Store was 60.5 MB. Siddiqui et al [43] found that most of the orthodontic mHealth apps on the Apple and Google app stores were <40 MB in size.

Considerations of app size are important due to their impact on the ease of downloading and using these apps. Research has consistently demonstrated that larger apps tend to prolong the downloading process and increase user burden during their use [71]. In addition, Haggag et al [24] underscored that those individuals with older smartphones or limited storage capacity reported significant challenges when engaging with mHealth apps.

The implication of this issue is particularly pronounced in low-resource settings, where access to more advanced smartphones and reliable internet connectivity for downloading and using these large apps is a persistent concern [46,72]. Addressing these logistical challenges and developing easily usable apps is essential in ensuring equitable access to mHealth apps, especially in Indian settings where resources are constrained.

### Price of Apps and In-App Purchases

We found that most of the HD apps were offered free of charge, aligning with observations made in prior studies [34]. For the minority of apps that did entail a cost, we identified that prices of apps were relatively higher on the Apple App Store compared to the Google Play Store. Furthermore, about 20% (125/588, 21.3%) of these apps featured in-app purchases on the Google Play Store. While it was noted that some paid health apps had shown increased user engagement [73], in general, both price and in-app purchases have been identified as potential barriers to the adoption and use of mHealth apps [40,74].

In low-resource settings, the appeal of mHealth apps often revolves around their potential to mitigate barriers to health care access, including the financial burdens of health care [75]. In this context, it is encouraging to observe that most HD apps are accessible to smartphone users free of cost. However, it is crucial to highlight that free apps may come with limited access to a wide range of interventions, comprehensive programs, and qualified health care professionals [75].

### Ratings and Installs: Google Play Store

The absence or scarcity of ratings has been linked to limited widespread use [47]. Dehling et al [47] found that apps with fewer ratings were less likely to be downloaded and used, possibly due to their diminished visibility in app stores relative to the number of ratings. Moreover, users tend to view apps with a greater number of ratings as more credible and trustworthy.

HD app installs on the Google Play Store vary widely, ranging from 1 to 1 million, with an average of approximately 2000 installs. It is important to note that while the number of installs provides an indication of app use, it does not necessarily reflect the number of active users.

### Rating-Scores and Reviews

Prior research indicates that app rating-scores do not strongly correlate with the clinical utility and validity of health apps [76]. In fact, rating-scores from app stores often diverge significantly from ratings provided by health care professionals and patients [77]. Despite this, rating-scores positively impact app downloads [76]. When users choose apps, rating-scores and reviews are frequently the sole available measures [77] that influence their decision to download the app [78]. Rating-scores and reviews also offer valuable user feedback, enabling app developers to enhance their apps [79].

In our study, most HD apps on both the Apple and Google app stores lacked rating-scores and reviews. However, among those apps with rating-scores, the majority received ratings >3 stars. Reviews, when present, typically ranged in number from 1 to 1000, with very few apps accumulating more than 1000 reviews. These findings suggest that active use and user engagement with HD apps in India are confined to only a fraction of the available apps.

### Length of Description

In our study, the average length of description for HD apps was around 1300 characters on the Apple App Store and around 1800 characters on the Google Play Store. The length of description in mHealth apps has been studied in previous research. In their study about psychiatric apps on Google Play Store, Pinheiro et al [74] reported that the app descriptions ranged from 75 to 3456 characters with an average of 1253 characters. Previous studies have indicated that the length of app descriptions in mHealth apps can influence their demand and install rates [71,79]. Typically, a more comprehensive and explanatory description tends to correlate positively with a higher number of installs. We discuss the correlation between the length of the description and other attributes further in the correlation analysis section below.

### OS Versions and Update Years

In our analysis, we observed that most HD apps available on both the Apple App Store and Google Play Store in India did not require the latest OS versions. This is significant because apps that demand newer OS versions may become inaccessible to users with older smartphones [24].

Furthermore, we found that most of the HD apps on the Apple App Store have received updates within the last few years. It is worth noting that the timing of the latest update has been linked to the quality of mHealth apps [76]. Specifically, apps updated within the previous 6 months tend to exhibit higher quality [76]. However, it is essential to highlight that app updates can sometimes lead to compatibility issues, impacting users’ ability to use these apps effectively. Such issues have been reported to hinder the use of certain mHealth apps [24].

### Release Years of Apps

The distribution of apps according to their release years (Figures 3E and 3F) reveals interesting observations. While Apple App Store HD apps peaked in 2021, Google Play Store HD apps reached their peak in 2020. However, it is essential to interpret this data cautiously, as it may not accurately reflect the actual number of HD apps released in the last 2 years. Several factors contribute to this disparity. First, our study builds upon Kaggle datasets acquired in October 2021 and June 2021, which might inherently carry a bias in terms of app release years due to variations in total app store coverage and the timing of data collection. Second, newer apps introduced to these platforms since June and October 2021 may have varying degrees of linkage to apps in our initial Kaggle datasets. This linkage relies on app similarity, defined by internal algorithms of the Google Play Store and the Apple App Store, and the presence of common developers, both of which are beyond our control.

Despite these potential limitations, it is important to note that our dataset remains extensive, encompassing >200,000 mHealth apps and >2000 HD apps across both platforms. While acknowledging these considerations, our study’s primary findings and insights into the landscape of mHealth apps, especially those related to HD, remain valid and valuable for researchers and stakeholders in the field.

### Correlation Analysis

This analysis sheds light on various aspects of HD apps, offering insights into factors influencing their downloads, user ratings, and overall dynamics.

We found that for HD apps on Google Play Store, the number of installs positively correlated with the number of ratings and reviews. Pinheiro et al [74] noted a similar strong correlation between the number of installs and ratings in psychiatry apps on the Google Play Store. Notably, they reported no significant correlation between installs and reviews [74]. Pinheiro et al [74] also reported a strong association between installs and low app prices, as well as installs and the presence of in-app purchases. Interestingly, these associations were not found in the context of HD apps in this study.

On both Apple App Store and Google Play Store, we found that app rating-score and description length were positively correlated. This suggests that longer descriptions tend to correspond to higher app rating-scores. We found that the release year of the apps was negatively associated with the number of reviews and rating-scores on both Apple App Store and Google Play Store, suggesting that newly released apps had a lower number of reviews and lower rating-scores, and older apps tended to have slightly higher rating-scores and reviews.

In addition, our analysis revealed a positive correlation between the size and price of HD apps on the Apple App Store, contrary to findings from the study by Fu [45]. Fu studied apps from all categories in Apple App Store, not only mHealth apps, and found that larger apps tend to be cheaper. In our analysis of HD apps, we found that larger apps, in general, were more expensive. The large size of such apps could be due to additional features offered.

### Clustering

We used LDA clustering techniques to identify and classify the different types of HD apps available in India. We found that about half of the apps were classified into the clinical cluster. This cluster had terms like doctor, disease, treatment, cardiac, and pain. This cluster can be used to conduct in-depth analysis and identify the numbers and characteristics of various clinical HD apps like those meant for teleconsultations, ordering medication, and health education.

Previous studies have used clustering techniques while studying apps from app stores. However, those apps were very different from our study. Fu [45] used numerical clustering methods on apps from the Apple App Store to group apps according to the number of ratings and user rating-score. Danahiswari et al [44] studied the Google Play Store apps and used k-means clustering to identify the differences in characteristics between apps that were attractive to users and those that were not. Both these studies were aimed toward app developers and making better apps.

Dehling et al [47] studied the security and privacy of mHealth apps on the Apple App Store and Google Play Store using clustering techniques. To the best of our knowledge, ours is the first study to use app descriptions to cluster and classify HD apps.

These clusters can be used in further research depending on one’s research focus. For example, researchers interested in preventive and public health aspects of HD apps may incorporate apps from the lifestyle and fitness clusters, whereas those seeking more clinical apps can concentrate solely on the clinical cluster.

Further analysis of app attributes within individual clusters showed that clinical apps were smaller in size. Among the largest (ie, >100 MB app size) apps, there were a few clinical apps. However, these were outliers, and most apps from the clinical cluster were much smaller. They also had shorter descriptions. Clinical cluster apps on the Google Play Store had a lower number of installs and rating-scores, and the lowest percentage of apps with in-app purchases. More detailed descriptions can perhaps help increase the installs of these apps.

Our analysis revealed key insights into the current landscape of health and medical apps in India. Language accessibility remains a significant barrier, with most apps not supporting regional languages. Despite the high prevalence of HD, there is a notable scarcity of relevant mHealth apps. The existing HD apps are generally large, creating challenges for users with limited storage or slow internet. While most HD apps are free, paid apps (ie, especially on the Apple App Store), and in-app purchases (ie, on the Google Play Store) can hinder accessibility. User engagement (ie, from the number of ratings and reviews, and rating) is in general low, possibly due to issues with app discoverability and perceived credibility. We found that longer app descriptions tend to correlate with higher ratings, enhancing user trust. Many apps support older OS versions, reducing technological barriers. However, frequent updates, while improving quality, can lead to compatibility issues. In recent years, there has been a peak in app releases. Correlation analysis showed that the year of release for the apps was closely linked to ratings and reviews. Apps that have been around for a while had more ratings and reviews, which can add to their perceived credibility. Clustering revealed that “clinical apps,” which focus on teleconsultation and health education, were prominent in number but had lower engagement. They had a lower number of installs, rating-scores, and shorter descriptions. Each of the clusters can be studied further in future research to obtain deeper insights.

### Limitations

We used a comprehensive approach and a thorough methodology in this study; however, it is important to acknowledge the limitations for a balanced interpretation of the findings.

A downside to using the Kaggle datasets is that any biases or inaccuracies in the original scraping process could have extended into our study. While we made diligent efforts to compile a comprehensive dataset of mHealth and HD apps, it is important to acknowledge the absence of a definitive list encompassing all available apps on both app stores to compare against. To enhance the reliability of future research based on this dataset, we propose a validation approach wherein our dataset can be cross-referenced with a carefully selected subset of apps that are unequivocally expected to be part of such a list.

While the keyword dictionaries cover an extensive list of relevant terms, they are not exhaustive as we may have missed some terms, leading to some missed HD apps. Furthermore, we used the tokenized text from the title, summary, and description of the apps as text data for NLP analysis, and we did not appraise the quality of the texts. Our method can be used as a first step to gather all these apps that claim to address specific health issues (eg, HD), which can then be scrutinized under a quality lens using the already existing tools, such as MARS (Mobile Application Rating Scale) [41,42].

Our analysis of HD apps exclusively focused on apps with English descriptions, excluding those with descriptions in an Indian language. However, it is important to note that apps with descriptions in an Indian language represent a minor fraction, 0.5% (548/108,945) in Google apps and 1.68% (1990/118,555) in Apple apps. Our aim was to offer a comprehensive overview of HD apps accessible in India. We highlight the scarcity of apps with descriptions in Indian languages and emphasize the importance of exploring these apps in future research.

The LDA model parameters were selected based on iterative testing; however, they might not be optimal for all types of health apps. They may need to be adjusted when these methods are replicated for other mHealth apps.

In our analysis, we used simple descriptive statistics and unsupervised clustering methods. More detailed statistical techniques and supervised topic modeling could yield deeper insights into the data. However, with this study, we aimed to establish our methods to create a database for health apps and analyze data to provide an overview of the HD apps available in India. We believe that these initial findings set the stage for more in-depth research, offering a baseline for future studies to build upon.

The results and models of this study are not validated with external datasets, limiting the robustness of the conclusions. However, we aimed to lay a strong foundation for future research by providing a comprehensive and replicable methodology. The code is available on GitHub, allowing other researchers to easily validate and extend our work with additional datasets, enhancing the credibility and broader applicability in future studies.

### Our Methods Versus Previous Methods

Studies on mHealth apps so far either looked at existing literature on specific apps or explored and analyzed a handful of apps by searching app stores manually. Manual scraping cannot help while mapping a vast number of apps. It only works for collecting data about a very specific health condition, and even then, it is time-taking.

Studies, such as the one by Dehling et al [47] have previously used similar scraping methods, but the regulations and designs of app stores have changed since then. Moreover, there is a limitation on the number of results one can obtain in both the app stores by searching with specific keywords. Our methods help increase the search range by using the “similar to” and “from the same developer” features on app stores and automate the process of collecting and screening metadata from a large number of apps.

### Future Research

In the future, this method can be used to compile a dynamic dataset of mHealth apps available in India at any given time, allowing for regular updates and enabling trend analysis over time. Expanding the analysis on the HD apps, one could look at apps concerning the major health priorities in India, such as the top 10 causes of death. Furthermore, an analysis of the users’ reviews using NLP techniques and sentiment analysis could help to understand the users’ views and challenges while adopting mHealth apps. Our study and methodology facilitate the selection of apps with particular characteristics for further qualitative evaluation with tools, such as MARS [41,42]. This enables a systematized understanding of the app quality, instead of depending on the blackbox methods used by the app stores for manual searching.

Our methods hold particular significance in mapping the landscape of mHealth apps, particularly in low-resource settings like India, where evidence on the rapidly evolving mHealth ecosystem is scarce. This study addresses a significant gap in the academic literature by offering a relatively user-friendly approach to access and analyze valuable metadata from mHealth apps. Importantly, our method is designed to be accessible to health researchers without extensive coding expertise, serving as an effective means to overcome this potential barrier. Researchers can readily adapt and replicate our approach to explore mHealth apps in various low-resource settings, leveraging geolocation and condition-specific keyword dictionaries to analyze apps tailored to address diverse health issues with targeted research inquiries.

### Conclusions

Our study introduced a robust method for collecting and analyzing metadata from a wide array of mHealth apps available in India through the Apple App Store and the Google Play Store. We revealed the limited representation of India’s linguistic diversity within the health and medical app landscape, evident from the negligible presence of Indian language apps. Despite the high prevalence and number of deaths caused by HD in India, we observed a disproportionate scarcity of mHealth apps dedicated to this crucial health issue, along with a lower level of user engagement, as indicated by reviews and app ratings. While most HD apps are financially accessible, uptake remains a challenge. Further research should focus on app quality assessment and factors influencing user adoption.

## Appendix

Multimedia Appendix 1 Language analysis on the Apple App Store and Google Play Store apps.

Multimedia Appendix 2 k-means clustering in heart disease apps on Apple App Store.

Multimedia Appendix 3 Language distribution in mobile health apps from the 2 app stores.

Multimedia Appendix 4 Average rating-scores, reviews, and installs by language for mobile health apps on the Google Play Store.

Multimedia Appendix 5 Keyword monograms and bigrams found in (selected) apps related to heart diseases.

Multimedia Appendix 6 Exploratory correlation analysis—Apple App Store app attributes.

Multimedia Appendix 7 Exploratory correlation analysis—Google Play Store app attributes.

Multimedia Appendix 8 Clustering and topic modeling via latent Dirichlet allocation for heart disease apps. PC: principal component.

Multimedia Appendix 9 Distribution of medical category apps in heart disease app clusters.

## Abbreviations

HD: heart disease
LDA: latent Dirichlet allocation
mHealth: mobile health
NLP: natural language processing
OS: operating systems

## References

[1] Lapão LV, Dussault G (2017). The contribution of eHealth and mHealth to improving the performance of the health workforce: a review. Policy Pract.

[2] (2018). mHealth: use of appropriate digital technologies for public health. World Health Organization.

[3] Chauhan P, Jadhav V, Gupta N (2021). COVID-19 pandemic and consumer adoption of digital healthcare in India: a qualitative study. Acad Mark Stud J.

[4] Madanian S, Parry DT, Airehrour D, Cherrington M (2019). mHealth and big-data integration: promises for healthcare system in India. BMJ Health Care Inform.

[5] Majumdar A, Kar SS, Palanivel C, Misra P (2015). mHealth in the prevention and control of non-communicable diseases in India: current possibilities and the way forward. J Clin Diagn Res.

[6] McCool J, Dobson R, Whittaker R, Paton C (2022). Mobile health (mHealth) in low- and middle-income countries. Annu Rev Public Health.

[7] Pai RR, Alathur S (2021). Bibliometric analysis and methodological review of mobile health services and applications in India. Int J Med Inform.

[8] Silva BM, Rodrigues JJ, de la Torre Díez I, López-Coronado M, Saleem K (2015). Mobile-health: a review of current state in 2015. J Biomed Inform.

[9] Rathore M Share of school children living in a family with a smartphone during COVID-19 pandemic in India as of August 2021, by region. Statista.

[10] Shangliao S Smartphone users in India 2010-2040. Statista.

[11] Holzman SB, Atre S, Sahasrabudhe T, Ambike S, Jagtap D, Sayyad Y, Kakrani AL, Gupta A, Mave V, Shah M (2019). Use of smartphone-based video directly observed therapy (vDOT) in tuberculosis care: single-arm, prospective feasibility study. JMIR Form Res.

[12] Jimenez G, Lum E, Car J (2019). Examining diabetes management apps recommended from a Google search: content analysis. JMIR Mhealth Uhealth.

[13] Kumar R, Das A (2021). The potential of mHealth as a game changer for the management of sickle cell disease in India. JMIR Mhealth Uhealth.

[14] Selvaraj SN, Sriram A (2022). The quality of Indian obesity-related mHealth apps: PRECEDE-PROCEED model-based content analysis. JMIR Mhealth Uhealth.

[15] Praveen D, Patel A, Raghu A, Clifford GD, Maulik PK, Mohammad Abdul A, Mogulluru K, Tarassenko L, MacMahon S, Peiris D (2014). SMARTHealth India: development and field evaluation of a mobile clinical decision support system for cardiovascular diseases in rural India. JMIR Mhealth Uhealth.

[16] Bassi A, Arfin S, John O, Jha V (2020). An overview of mobile applications (apps) to support the coronavirus disease 2019 response in India. Indian J Med Res.

[17] Jaiswal S, Kaur N, Bhalla M, Sharma VS, Gupta R, Chaudhary S (2020). A survey on knowledge and awareness among Indian citizens toward application to fight against COVID-19: a cross-sectional study. J Adv Clin Res Insights.

[18] Kaur M, Kaur H, Rathi S, Ashwitha M, Joanna J, Reddy S, Idris B, Myrtle P, Kandamuru S, Fatima S, Joshi A (2022). Apps on Google Play Store to assist in self-management of hypertension in Indian context: features analysis study. Mhealth.

[19] Kodali PB, Das S (2021). Acceptance of mHealth technologies among auxiliary; nurse midwives in Andhra Pradesh, India: a mixed method study. Med Sci.

[20] Bommakanti KK, Smith LL, Liu L, Do D, Cuevas-Mota J, Collins K, Munoz F, Rodwell TC, Garfein RS (2020). Requiring smartphone ownership for mHealth interventions: who could be left out?. BMC Public Health.

[21] Sinha Deb K, Tuli A, Sood M, Chadda R, Verma R, Kumar S, Ganesh R, Singh P (2018). Is India ready for mental health apps (MHApps)? A quantitative-qualitative exploration of caregivers' perspective on smartphone-based solutions for managing severe mental illnesses in low resource settings. PLoS One.

[22] Singh S, Sharma P, Ghimire P, Shrestha R, Gnanavel S (2023). Assessment of app store description and privacy policy to explore ethical and safety concerns associated with the use of mental health apps for depression. Indian J Psychol Med.

[23] Huang Z, Lum E, Jimenez G, Semwal M, Sloot P, Car J (2019). Medication management support in diabetes: a systematic assessment of diabetes self-management apps. BMC Med.

[24] Haggag O, Grundy J, Abdelrazek M, Haggag S (2022). A large scale analysis of mHealth app user reviews. Empir Softw Eng.

[25] Garnett A, Northwood M, Ting J, Sangrar R (2022). mHealth interventions to support caregivers of older adults: equity-focused systematic review. JMIR Aging.

[26] Bassi A, John O, Praveen D, Maulik PK, Panda R, Jha V (2018). Current status and future directions of mHealth interventions for health system strengthening in India: systematic review. JMIR Mhealth Uhealth.

[27] Coulon SM, Monroe CM, West DS (2016). A systematic, multi-domain review of mobile smartphone apps for evidence-based stress management. Am J Prev Med.

[28] Dugas M, Gao GG, Agarwal R (2020). Unpacking mHealth interventions: a systematic review of behavior change techniques used in randomized controlled trials assessing mHealth effectiveness. Digit Health.

[29] Griswold D, Rubiano AM (2022). Role of mHealth applications for emergency medical system activation in reducing mortality in low-income and middle-income countries: a systematic review protocol. BMJ Open.

[30] Ming LC, Untong N, Aliudin NA, Osili N, Kifli N, Tan CS, Goh KW, Ng PW, Al-Worafi YM, Lee KS, Goh HP (2020). Mobile health apps on COVID-19 launched in the early days of the pandemic: content analysis and review. JMIR Mhealth Uhealth.

[31] Jahnel T, Dassow HH, Gerhardus A, Schüz B (2022). The digital rainbow: digital determinants of health inequities. Digit Health.

[32] DeWitt A, Kientz J, Coker TR, Liljenquist K (2022). mHealth technology design and evaluation for early childhood health promotion: systematic literature review. JMIR Pediatr Parent.

[33] Daley BJ, Ni'Man M, Neves MR, Bobby Huda MS, Marsh W, Fenton NE, Hitman GA, McLachlan S (2022). mHealth apps for gestational diabetes mellitus that provide clinical decision support or artificial intelligence: a scoping review. Diabet Med.

[34] Balsari S, Fortenko A, Blaya JA, Gropper A, Jayaram M, Matthan R, Sahasranam R, Shankar M, Sarbadhikari SN, Bierer BE, Mandl KD, Mehendale S, Khanna T (2018). Reimagining health data exchange: an application programming interface-enabled roadmap for India. J Med Internet Res.

[35] Stach M, Kraft R, Probst T, Messner EM, Terhorst Y, Baumeister H, Schickler M, Reichert M, Sander LB, Pryss R (2020). Mobile health app database - a repository for quality ratings of mHealth apps. Proceedings of the 33rd International Symposium on Computer-Based Medical Systems.

[36] Hamrioui S, González JH, Castillo G, de la Torre-Diez I, Lopez-Coronado M (2018). Development and QoE evaluation of an iOS mHealth app for self-controlling and education of patients with heart diseases. Proceedings of the 13th Iberian Conference on Information Systems and Technologies.

[37] Braghin C, Cimato S, Della Libera A (2018). Are mHealth apps secure? A case study. Proceedings of the 42nd Annual Computer Software and Applications Conference.

[38] Sampat BH, Prabhakar B (2017). Privacy risks and security threats in mHealth apps. J Int Technol Inf Manag.

[39] Kao CK, Liebovitz DM (2017). Consumer mobile health apps: current state, barriers, and future directions. PM R.

[40] König LM, Attig C, Franke T, Renner B (2021). Barriers to and facilitators for using nutrition apps: systematic review and conceptual framework. JMIR Mhealth Uhealth.

[41] Stoyanov SR, Hides L, Kavanagh DJ, Zelenko O, Tjondronegoro D, Mani M (2015). Mobile app rating scale: a new tool for assessing the quality of health mobile apps. JMIR Mhealth Uhealth.

[42] Stoyanov SR, Hides L, Kavanagh DJ, Wilson H (2016). Development and validation of the user version of the mobile application rating scale (uMARS). JMIR Mhealth Uhealth.

[43] Siddiqui NR, Hodges S, Sharif MO (2019). Availability of orthodontic smartphone apps. J Orthod.

[44] Danahiswari HC, Nurpratama YF, Kartika DS (2022). Application cluster analysis on the google play store using the K-Means method. Int J Comp Net Sec Inf Syst.

[45] Fu B (2020). Characteristics classification of mobile apps on apple store using clustering. J Data Anal Inf Process.

[46] Xu W, Liu Y (2015). mHealthApps: a repository and database of mobile health apps. JMIR Mhealth Uhealth.

[47] Dehling T, Gao F, Schneider S, Sunyaev A (2015). Exploring the far side of mobile health: information security and privacy of mobile health apps on iOS and Android. JMIR Mhealth Uhealth.

[48] Tsinaraki C, Mitton I, Minghini M, Micheli M, Kotsev A, Hernandez Quiros L, Spinelli F, Dalla Benetta A, Schade S (2020). Analysing mobile apps that emerged to fight the COVID-19 crisis. European Commission.

[49] GBD 2019 Diseases Injuries Collaborators (2020). Global burden of 369 diseases and injuries in 204 countries and territories, 1990-2019: a systematic analysis for the Global Burden of Disease Study 2019. Lancet.

[50] Demner-Fushman D, Chapman WW, McDonald CJ (2009). What can natural language processing do for clinical decision support?. J Biomed Inform.

[51] Juhn Y, Liu H (2020). Artificial intelligence approaches using natural language processing to advance EHR-based clinical research. J Allergy Clin Immunol.

[52] Kumar S, Singh M (2019). A novel clustering technique for efficient clustering of big data in Hadoop Ecosystem. Big Data Min Anal.

[53] Chen X, Xie H, Wang FL, Liu Z, Xu J, Hao T (2018). A bibliometric analysis of natural language processing in medical research. BMC Med Inform Decis Mak.

[54] Iroju OG, Olaleke JO (2015). A systematic review of natural language processing in healthcare. Int J Inf Technol Comput Sci.

[55] Prakash G, Koshy J Google Play store apps. kaggle.

[56] Prakash G Apple AppStore apps. kaggle.

[57] Olano F google-play-scraper (v9.1.1). npm Inc.

[58] Olano F app-store-scraper (v0.17.0). npm Inc.

[59] Leeson W, Resnick A, Alexander D, Rovers J (2019). Natural language processing (NLP) in qualitative public health research: a proof of concept study. Int J Qual Methods.

[60] Nadkarni PM, Ohno-Machado L, Chapman WW (2011). Natural language processing: an introduction. J Am Med Inform Assoc.

[61] (2020). Myocardial ischemia. National Library of Medicine.

[62] pandas-dev/pandas: pandas. zenodo.

[63] Hunter JD (2007). Matplotlib: a 2D graphics environment. Comput Sci Eng.

[64] Bird S, Edward L, Ewan K (2009). Natural Language Processing with Python: Analyzing Text with the Natural Language Toolkit.

[65] Lopez F fedelopez77/langdetect. GitHub.

[66] Mueller AC Wordcloud. GitHub.

[67] Mabey B bmabey/pyLDAvis. GitHub.

[68] Prizak R, Dubbala K General · KeerthiDubbala/mHealth-apps-in-India. GitHub.

[69] Mechael P, Batavia H, Kaonga N, Searle S, Kwan A, Goldberger A, Fu L, Ossman J (2010). Barriers and gaps affecting mHealth in low- and middle-income countries: policy white paper. Center for Global Health and Economic Development, The Earth Institute at Columbia University. University of California.

[70] Bassi A (2016). mHealth interventions for health system strengthening in India. George Institute for Global Health.

[71] Pereira-Azevedo N, Osório L, Cavadas V, Fraga A, Carrasquinho E, Cardoso de Oliveira E, Castelo-Branco M, Roobol MJ (2016). Expert involvement predicts mHealth app downloads: multivariate regression analysis of urology apps. JMIR Mhealth Uhealth.

[72] Wallis L, Blessing P, Dalwai M, Shin SD (2017). Integrating mHealth at point of care in low- and middle-income settings: the system perspective. Global Health Action.

[73] Sharma S, Barnett KG, Maypole JJ, Mishuris RG (2022). Evaluation of mHealth Apps for Diverse, Low-Income Patient Populations: Framework Development and Application Study. JMIR Form Res.

[74] Pinheiro M, Serra M, Pereira-Azevedo N (2019). Predictors of the number of installs in psychiatry smartphone apps: systematic search on app stores and content analysis. JMIR Ment Health.

[75] Grundy Q (2022). A review of the quality and impact of mobile health apps. Annu Rev Public Health.

[76] Wisniewski H, Liu G, Henson P, Vaidyam A, Hajratalli NK, Onnela JP, Torous J (2019). Understanding the quality, effectiveness and attributes of top-rated smartphone health apps. Evid Based Ment Health.

[77] Hudson G, Negbenose E, Neary M, Jansli SM, Schueller SM, Wykes T, Jilka S (2022). Comparing professional and consumer ratings of mental health apps: mixed methods study. JMIR Form Res.

[78] How ratings and reviews affect consumers decision to download apps. Business of Apps.

[79] Galetsi P, Katsaliaki K, Kumar S, Ferguson M (2021). What affects consumer behavior in mobile health professional diagnosis applications. Decis Sci.

