# Supplementary figures and images for: Exploring Heart Disease–Related mHealth Apps in India: Systematic Search in App Stores and Metadata Analysis
Source: J Med Internet Res. 2025 Mar 10;27:e53823. doi: 10.2196/53823 (PMC11933765; doi:10.2196/53823)

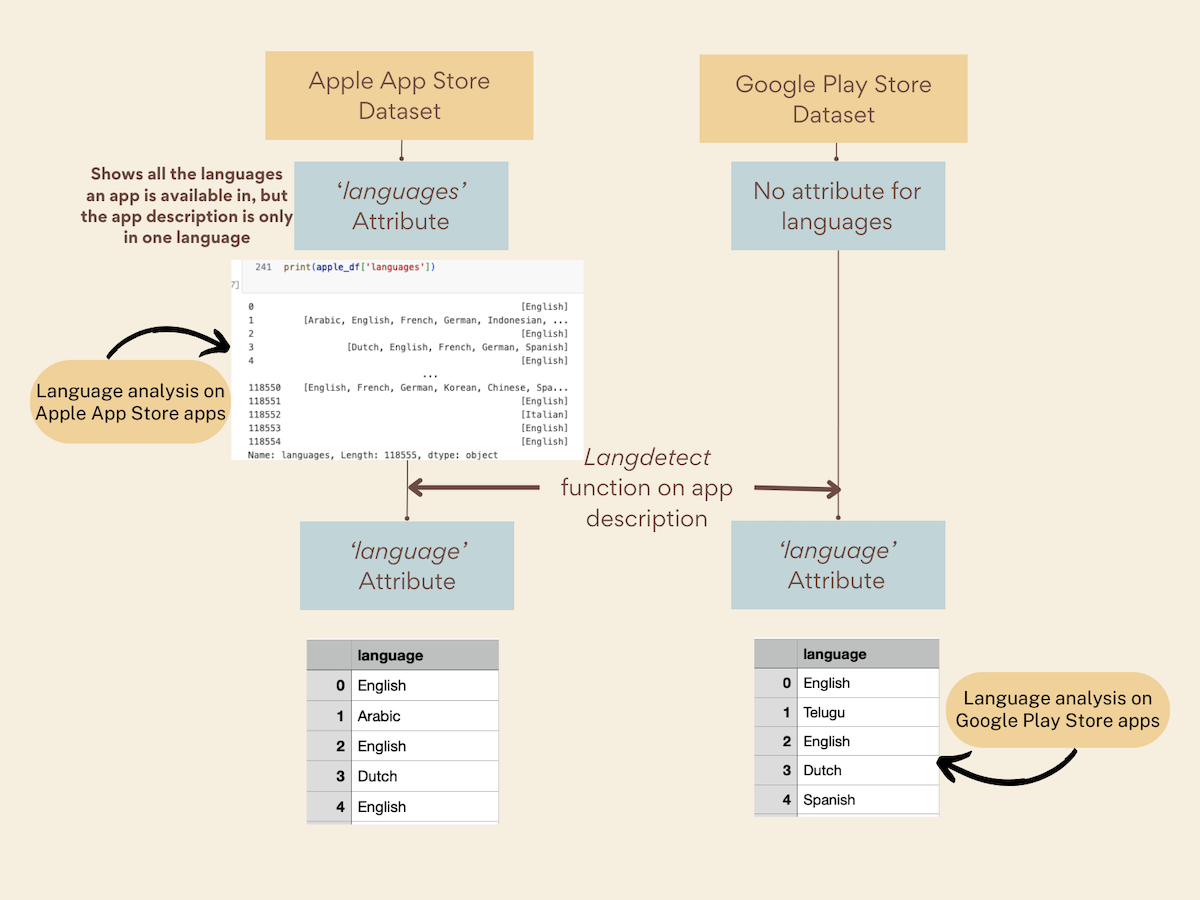

Supplement: Multimedia Appendix 1 [file jmir_v27i1e53823_app1.png]

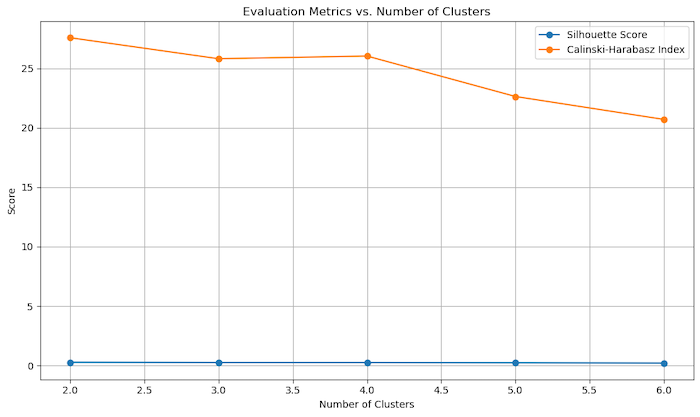

Supplement: Multimedia Appendix 2 [file jmir_v27i1e53823_app2.png]

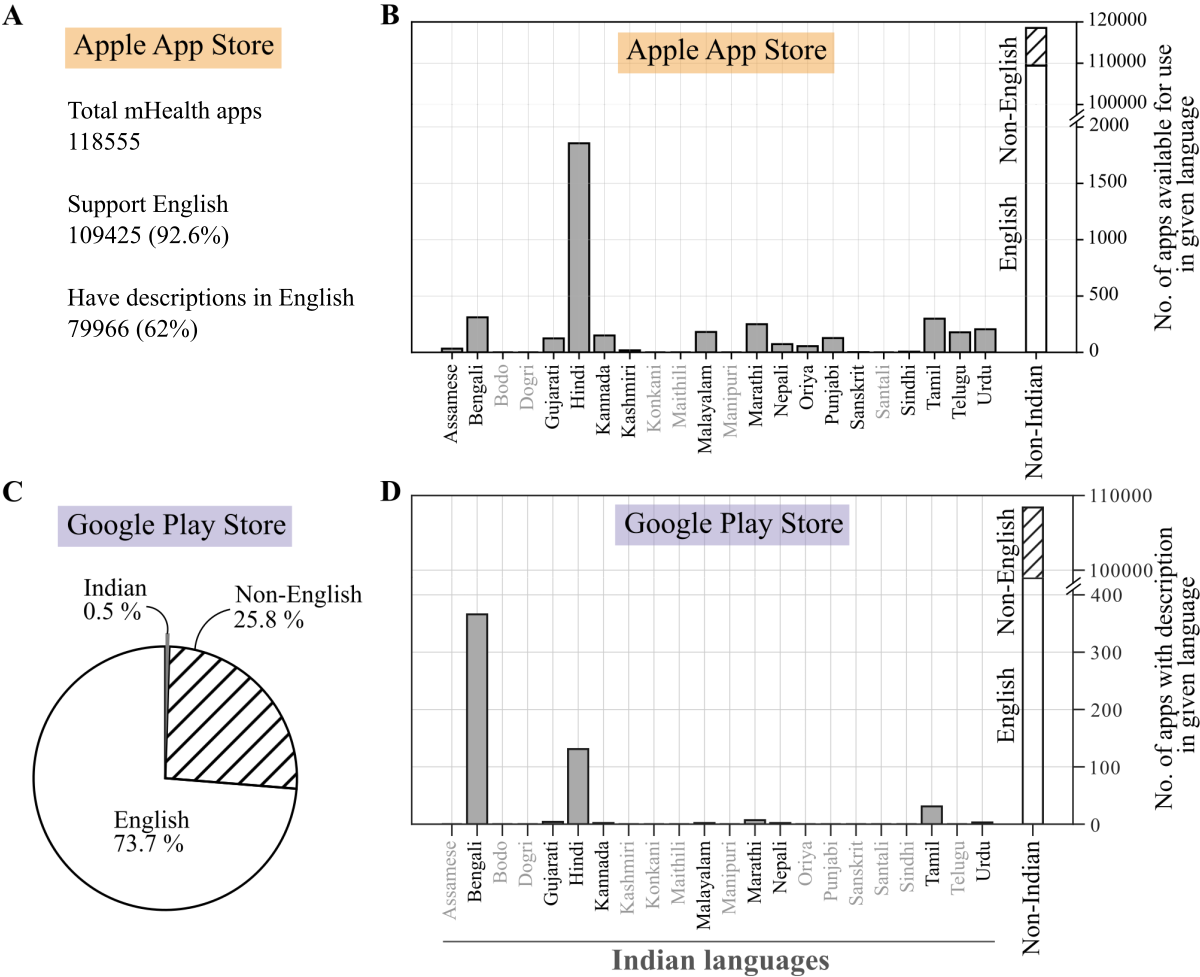

Supplement: Multimedia Appendix 3 [file jmir_v27i1e53823_app3.png]

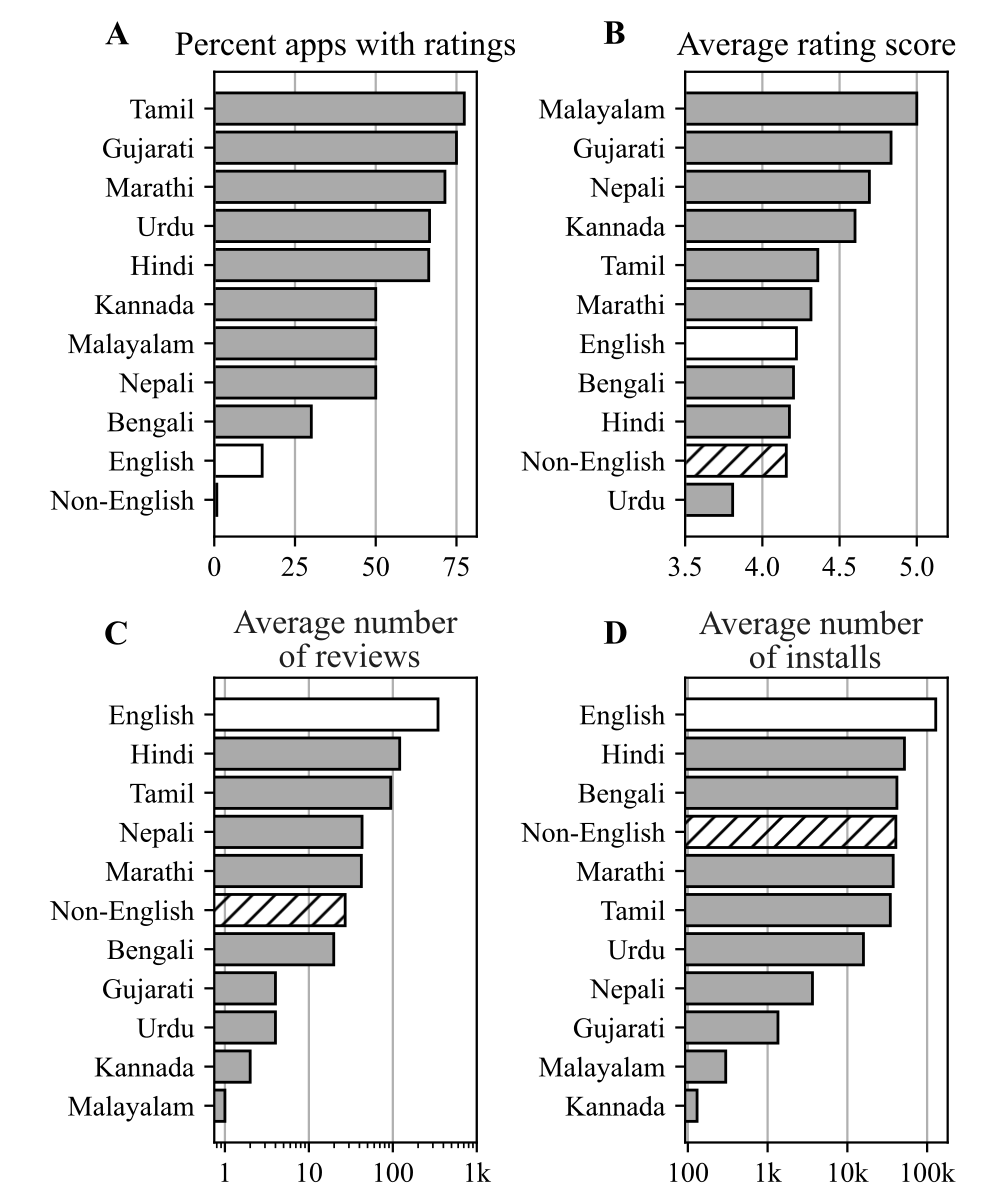

Supplement: Multimedia Appendix 4 [file jmir_v27i1e53823_app4.png]

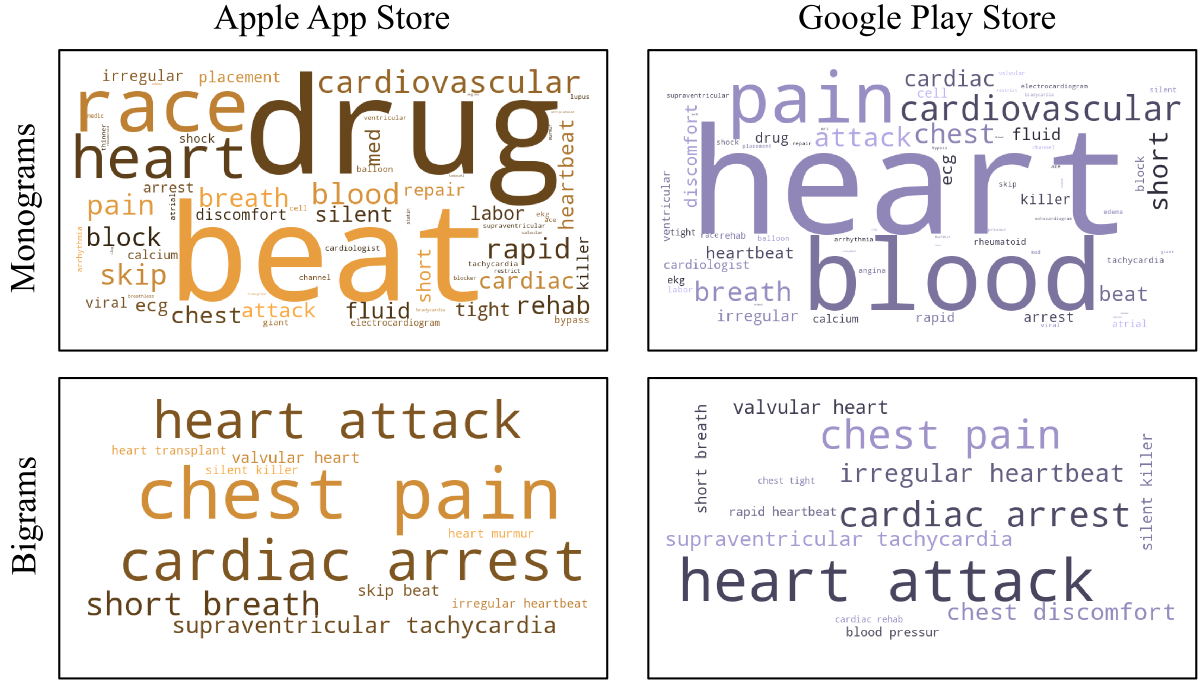

Supplement: Multimedia Appendix 5 [file jmir_v27i1e53823_app5.png]

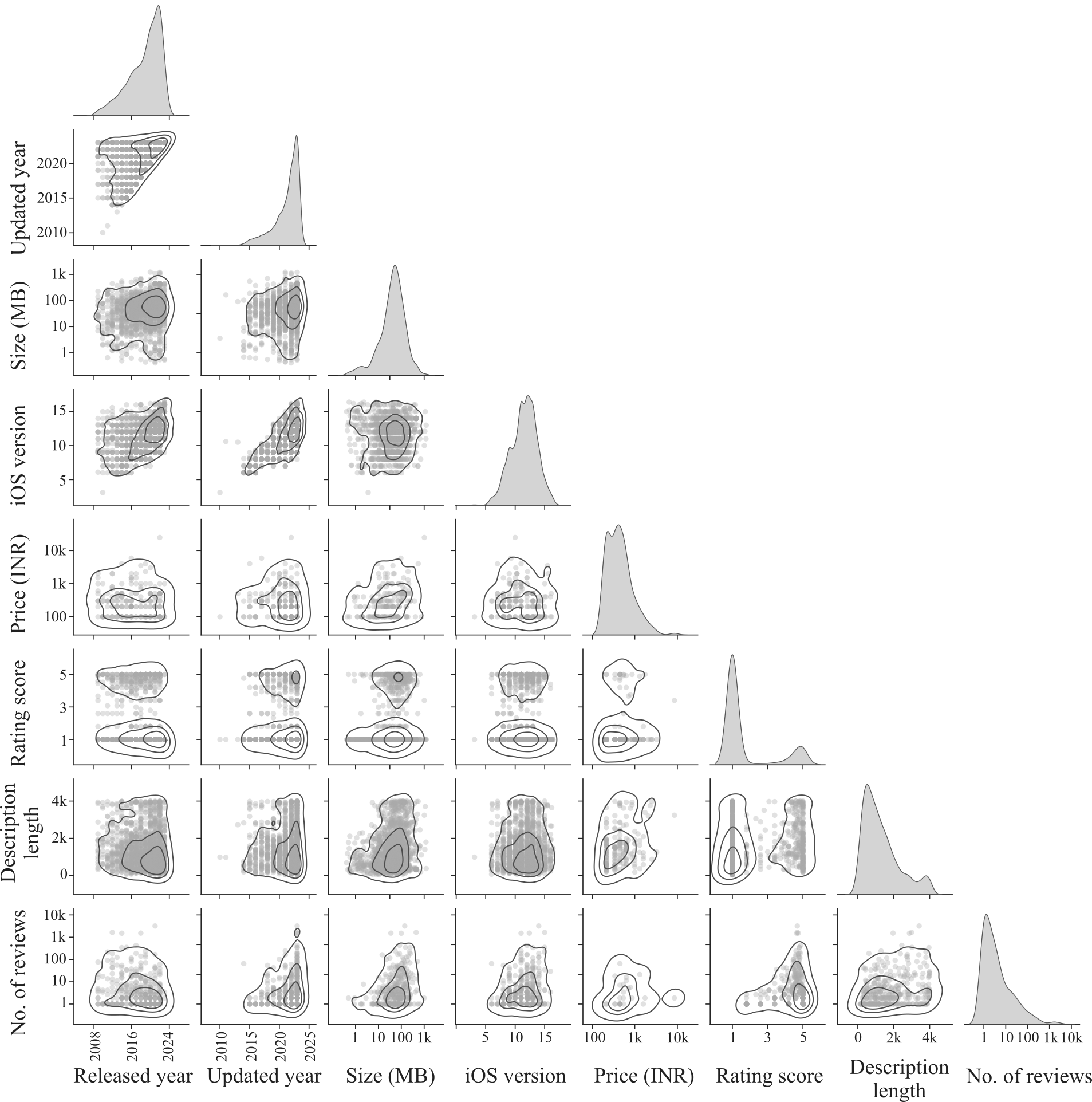

Supplement: Multimedia Appendix 6 [file jmir_v27i1e53823_app6.png]

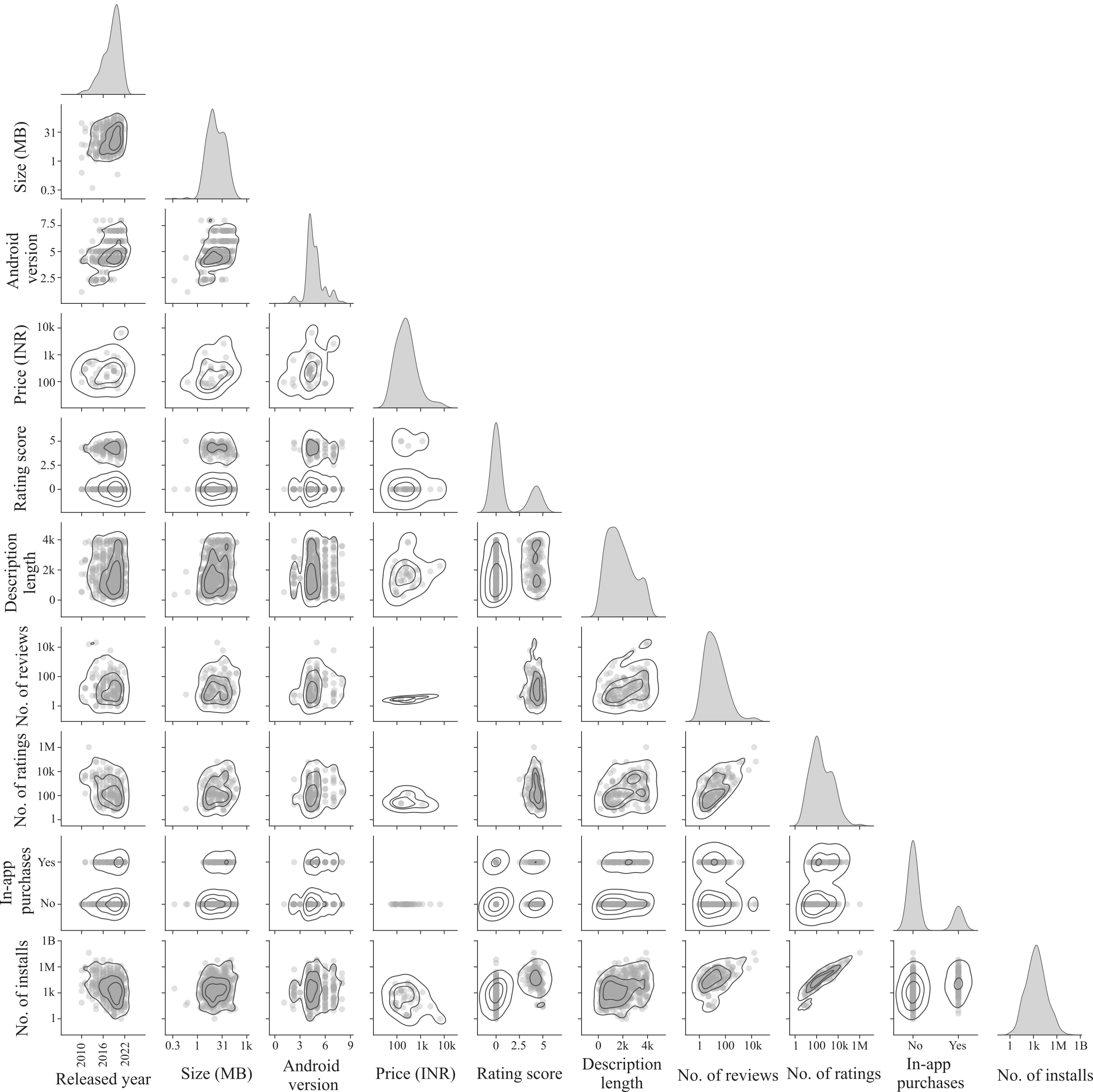

Supplement: Multimedia Appendix 7 [file jmir_v27i1e53823_app7.png]

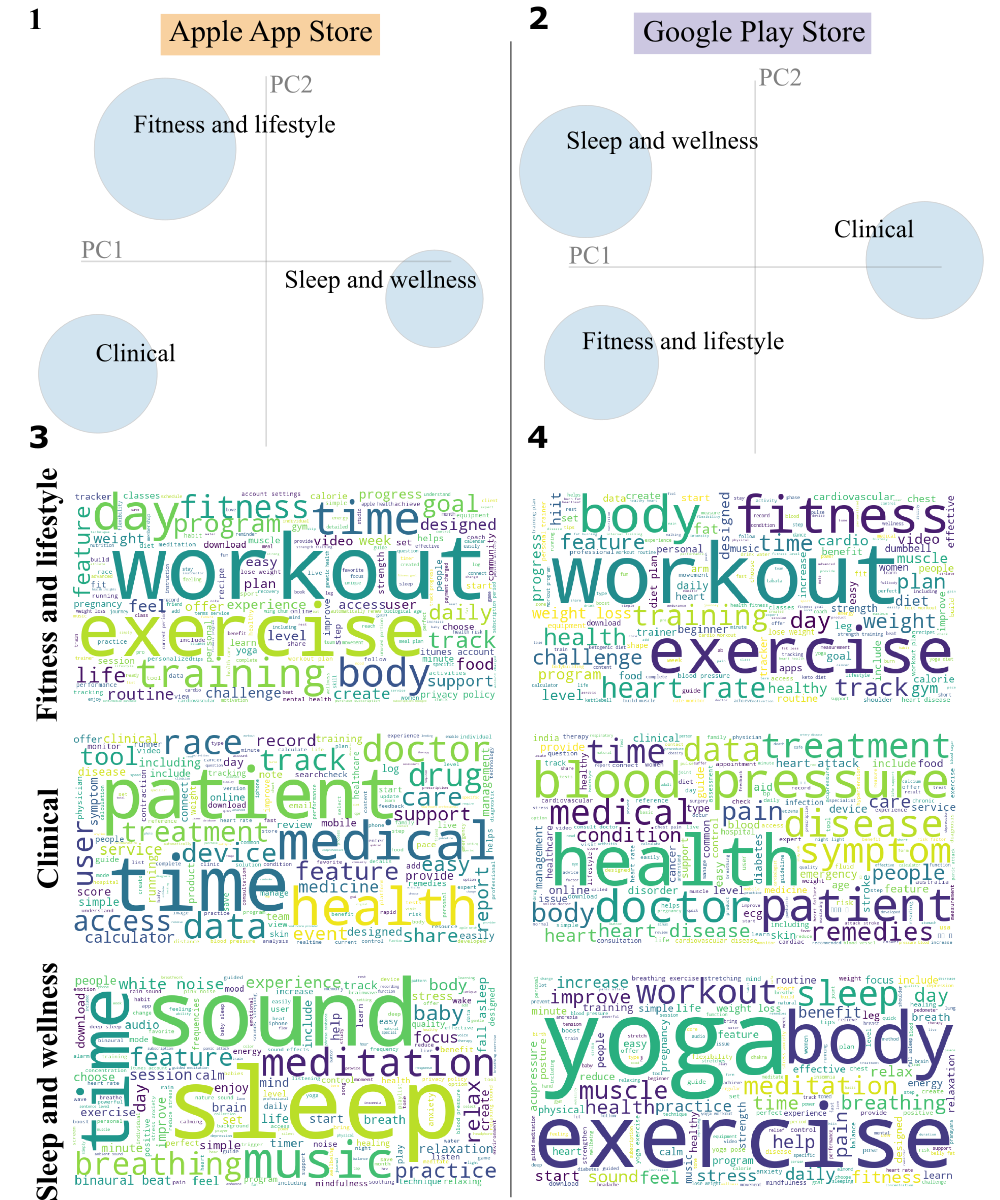

Supplement: Multimedia Appendix 8 [file jmir_v27i1e53823_app8.png]

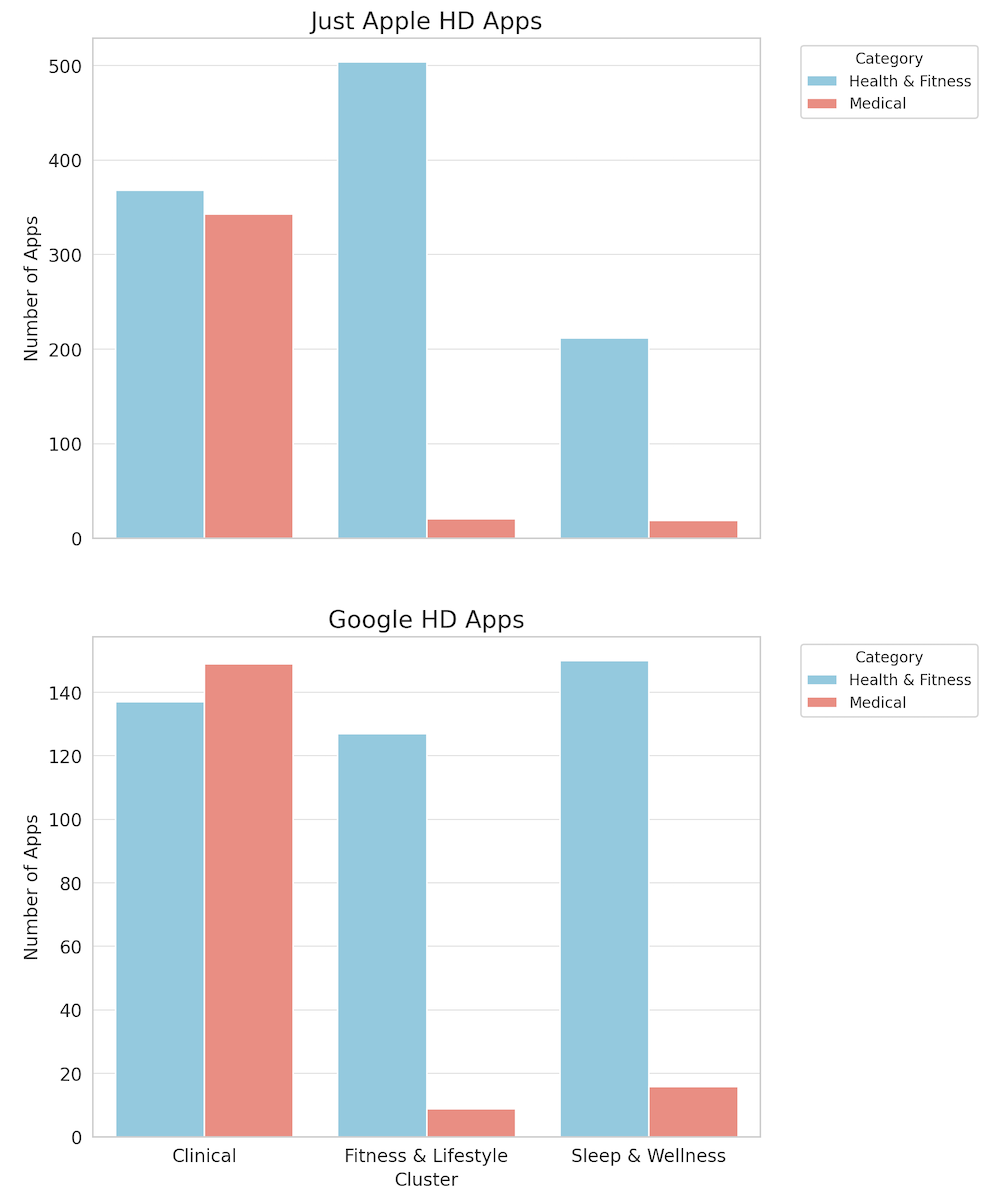

Supplement: Multimedia Appendix 9 [file jmir_v27i1e53823_app9.png]
